# Central-Pacific El Niño-Southern Oscillation less predictable under greenhouse warming

**DOI:** 10.1038/s41467-024-48804-1

**Published:** 2024-05-22

**Authors:** Hui Chen, Yishuai Jin, Zhengyu Liu, Daoxun Sun, Xianyao Chen, Michael J. McPhaden, Antonietta Capotondi, Xiaopei Lin

**Affiliations:** 1 Frontier Science Center for Deep Ocean Multispheres and Earth System (FDOMES) and Physical Oceanography Laboratory, Ocean University of China, Qingdao, China; 2 Laoshan Laboratory, Qingdao, China; 3 Atmospheric Science Program, Department of Geography, The Ohio State University, Columbus, OH USA; 4 School of Geography Science, Nanjing Normal University, Nanjing, China; 5 National Oceanic and Atmospheric Administration/Pacific Marine Environmental Laboratory, Seattle, WA USA; 6 Physical Sciences Laboratory, NOAA, Boulder, CO USA

**Keywords:** Projection and prediction, Physical oceanography

## Abstract

El Niño-Southern Oscillation (ENSO) is the dominant mode of interannual climate variability in the tropical Pacific, whose nature nevertheless may change significantly in a warming climate. Here, we show that the predictability of ENSO may decrease in the future. Across the models in the Coupled Model Intercomparison Project Phase 6 (CMIP6), we find a robust decrease of the persistence and predictability for the Central Pacific (CP) ENSO under global warming, notably in passing through the boreal spring. The strength of spring predictability barrier will be increased by 25% in the future. The reduced predictability of CP ENSO is caused by the faster warming over surface ocean in tropical Pacific and, in turn, the enhanced thermodynamical damping rate on CP ENSO in response to global warming. In contrast, the predictability of Eastern Pacific ENSO will not change. Our results suggest that future greenhouse warming will make the prediction of CP ENSO more challenging, with far-reaching implications on future climate predictions.

## Abstract (web summary)

This study shows that the predictability of Central Pacific ENSO will be suppressed in response to global warming, driven by the faster warming over surface ocean in tropical Pacific and a consequent enhanced thermodynamical damping.

## Introduction

El Niño-Southern Oscillation (ENSO) is the dominant mode of interannual climate variability in the tropical Pacific^1–3^. ENSO influence has been detected worldwide, on extreme weather events, ecosystems, and agriculture around the world^2,4–7^. ENSO events are typically classified into the Eastern-Pacific (EP) type (maximum warming/cooling appears in the equatorial eastern Pacific) and Central Pacific (CP) type (maximum warming/cooling appears in the equatorial central Pacific) because of their distinct spatial and temporal characteristics, and climate impacts^8–10^.

Extensive studies have investigated the impacts of future climate change on ENSO characteristics in different warming scenarios^11–14^. A robust intensifying trend of the magnitude of sea surface temperature anomaly (SSTA) associated with ENSO variability has been projected in the next century under various emission scenarios^13^. ENSO’s response to global warming has far-reaching implications for the global climate system^15,16^, including slowing the rate of future mid-latitude Southern Ocean warming^17^ and accelerating Antarctic shelf ocean warming^18^. On the other hand, extratropical climate change may also be an important factor for modulating the tropical climate (e.g., Walker circulation)^19^.

In spite of these studies, much less attention has been paid to the change of ENSO predictability in a warming climate, leaving a fundamental question wide open: How will greenhouse warming affects ENSO predictability in the future? A few studies have shown a decrease in ENSO predictability in recent decades^20,21^. However, due to the relatively short observations and the possible modulation by internal multi-decadal climate variability^22,23^, it remains unclear whether this transient reduction of predictability is induced by the warming climate. In an equilibrated future warming climate, the 6-month persistence of ENSO is slightly reduced^24^. However, ENSO predictability depends not only on the SSTA persistence, but also on other factors, notably the cross-correlation between SSTA and subsurface ocean heat content in the equatorial Pacific^25,26^. Therefore, both SSTA persistence and subsurface ocean heat content should be studied to fully evaluate ENSO predictability. Moreover, ENSO predictability may also be affected by extratropical processes^12,27,28^. Notably, the North Pacific Meridional Mode (NPMM) is expected to strengthen in a warming climate and thus become a more influential precursor of ENSO, enhancing its predictability^12,28^. The inter-basin interactions from the Atlantic and Indian Oceans, as well as sub-seasonal variability, such as the Madden-Julian Oscillation, can also affect the ENSO dynamics^29,30^ and its predictability^31–34^. As such, the change of ENSO predictability under global warming remains unknown. Finally, ENSO predictability is closely associated with the spring predictability barrier (SPB), which refers to the sharp drop of ENSO prediction skill across the boreal spring season^35^. The SPB still remains an outstanding issue in current ENSO prediction^36^ and its response of the ENSO SPB to global warming has not been studied.

Here, using outputs from the latest climate models participating in the Coupled Model Intercomparison Project Phase 6 (CMIP6), we find a robust decrease of predictability of CP ENSO, associated with the increase of the SPB strength, suggesting less predictable CP ENSO in a future warming climate. In contrast, there is no significant change for EP ENSO predictability.

## Results

### Enhanced persistence barrier of CP ENSO under greenhouse warming

**Fig. 1:**
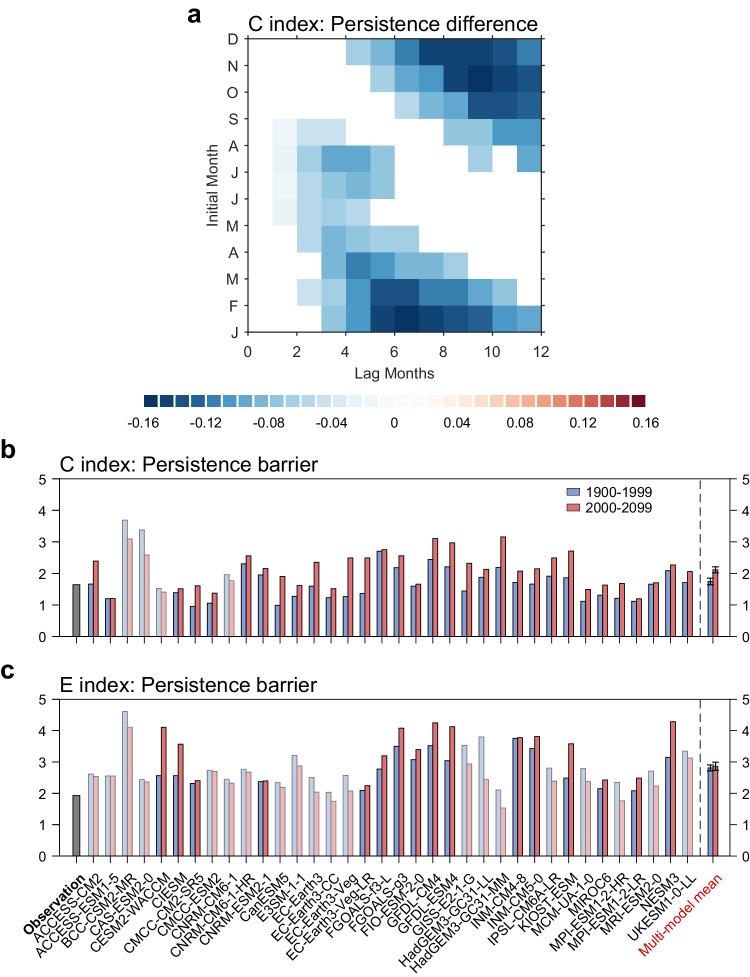
Projected changes in persistence of El Niño-Southern Oscillation. **a** The change in multi-model mean persistence map of Central-Pacific (CP) El Niño-Southern Oscillation (ENSO) between future (2000–2099) and present-day (1900–1999) climates. Only the difference (future minus present-day) exceeding 95% confidence level is shown in the figure (see bootstrap test in Methods). **b**, **c** Comparison of (**b**) CP and (**c**) Eastern-Pacific (EP) ENSO persistence barrier strength over present-day (blue bars) and future (red bars). Error bars are calculated as 1.0 standard deviation of 10,000 inter-realizations of a bootstrap method (see bootstrap test in Methods). Models that are opposite to the multi-model mean result are marked in white shading.

We first examine the persistence of ENSO, because model persistence can be compared directly with present observations, and because persistence is one major contributor to predictability, with a higher persistence implying more information from the previous month for the prediction of the next month^35^. Since CMIP6 can reproduce the observational features of the CP and EP ENSO as well as the seasonal evolution of their persistence (Supplementary Note 1, Supplementary Figs. 1 and 2), we first analyze the persistence in CMIP6. Here we use C and E indices to represent the CP and EP types of ENSO, which are defined as $\left({{{{{\rm{PC}}}}}}1+{{{{{\rm{PC}}}}}}2\right)/\sqrt{2}$ and $\left({{{{{\rm{PC}}}}}}1-{{{{{\rm{PC}}}}}}2\right)/\sqrt{2}$ respectively, because they are often used to study ENSO diversity under global warming^11,37^. We begin by evaluating the change of the year-round SSTA persistence of ENSO between the present-day (1900–1999) and future (2000–2099) periods. In total, 33 of the 36 models (92%) show a decreased year-round persistence for the CP ENSO in the future period relative to the present-day (red vs. blue bars in Supplementary Fig. 3a). The multi-model mean of the CP ENSO persistence is decreased significantly at the 95% confidence level according to a bootstrap test (see Methods). This decrease of year-round persistence is caused predominantly by that for the target month of boreal spring, or the spring persistence barrier of ENSO. This can be seen in the change of the multi-model mean of the seasonal evolution of the persistence of CP ENSO, which shows a significant (over 95% level) reduction for the target months around the boreal spring to summer (Fig. 1a), corresponding to a stronger spring persistence barrier in the future climate (Fig. 1b). For individual models, 32 of the 36 models (89%) simulate a significantly increased spring persistence barrier strength (see Methods) of CP ENSO in the future period (Fig. 1b). The multi-model mean increase in the spring persistence barrier strength of CP ENSO will be 21% in the future, which is significant at more than 95% confidence level according to a bootstrap test. The correspondence of the reduction of year-round persistence with the strength of the spring persistence barrier across models can also be seen in the cross-model scatter diagram (Supplementary Fig. 4a, b). In contrast, for the EP ENSO, there is no robust change in either the year-round persistence (Supplementary Fig. 3b), or the spring persistence barrier strength (Fig. 1c). The conclusion remains unchanged when we use other definitions of CP and EP ENSO (such as the NCP and NEP index^38^), or we use the subset of 28 CMIP6 models that can simulate ENSO nonlinearity (i.e., CP and EP ENSO^37^), both for the year-round persistence and spring persistence barrier (Supplementary Fig. 5, 6). We also find that the timing of the persistence barrier (see Methods) occurs one month earlier with global warming (Supplementary Fig. 7a). Since part of the ENSO predictability is contributed by the SSTA persistence^25^, the reduced persistence and increased strength of spring persistence barrier of CP ENSO in a warming climate should contribute to reduced predictability.

### Increased SPB of CP ENSO under greenhouse warming

**Fig. 2:**
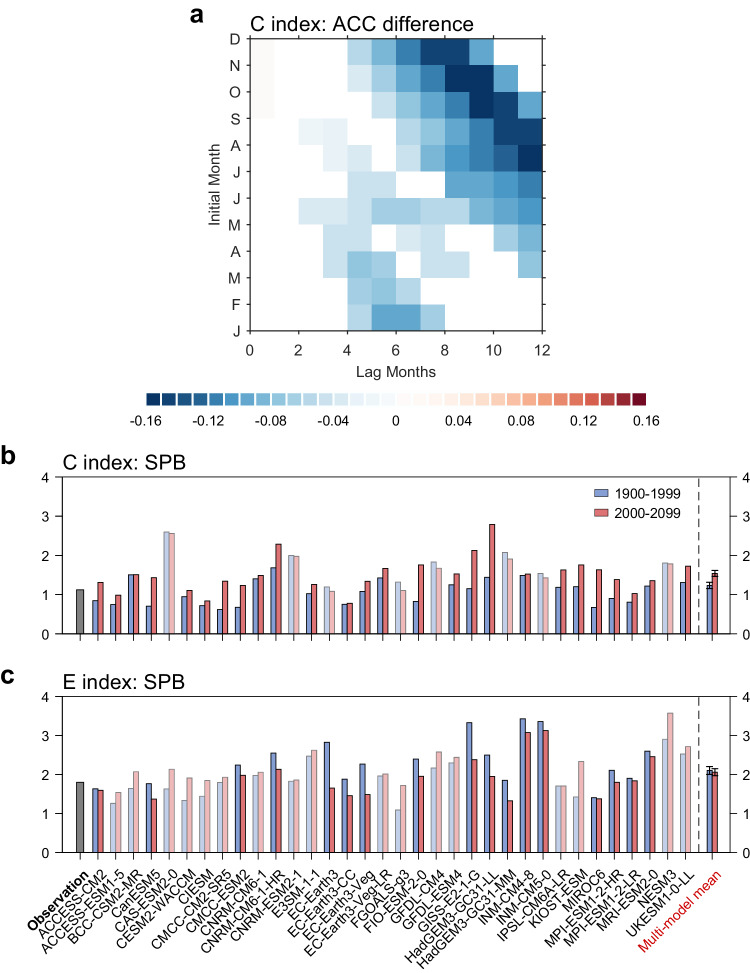
Projected change in spring predictability barrier of El Niño-Southern Oscillation. **a** The change in multi-model-mean anomaly correlation coefficient (ACC) map obtained from LIM for central Pacific (CP) El Niño-Southern Oscillation (ENSO) between future (2000–2099) and present-day (1900–1999) climates. Only the difference (future minus present-day) exceeding 95% confidence level is shown in the figure (see bootstrap test in Methods). **b**, **c** Comparison of (**b**) CP and (**c**) eastern-Pacific (EP) ENSO spring predictability barrier (SPB) strength predicted by linear inverse model (LIM) over present-day (blue bars) and future (red bars). Error bars are calculated as 1.0 standard derivation of 10,000 inter-realizations of a bootstrap method (see bootstrap test in Methods). Models that are opposite to the multi-model mean result are marked in white shading.

We then assess ENSO predictability and SPB using a Linear Inverse Model (LIM) of the tropical Pacific SST. LIM has been used extensively in the study of tropical Pacific SST variability and has shown prediction skill for real world ENSO largely comparable with dynamic models^39^. Here, LIM is used to further investigate ENSO predictability under global warming in CMIP6 models. Under the LIM framework (see Methods), we find a significant decrease in the anomaly correlation coefficient (ACC) of CP ENSO prediction in both the year-round ACC (Supplementary Fig. 8a) and for the target month of boreal spring (Fig. 2a). Across individual models, 26 out of 34 models (76%) project an increased strength in the SPB for CP ENSO significant at the 95% confidence level (Fig. 2b). The multi-model mean increase in the SPB strength of CP ENSO will be 25% in the future, which is significant at more than 95% confidence level according to a bootstrap test. In contrast, no significant change in the prediction ACC is detected for EP ENSO (Supplementary Fig. 8b and Fig. 2c). The largely consistent reduction of year-round ACC with the strength of SPB across models can also be seen in the cross-model scatter diagram (Supplementary Fig. 4c, d). These results suggest that CP ENSO predictability will likely decrease, mainly due to the difficulty of prediction to pass through the boreal spring in a future warming climate. The conclusion remains unchanged when we use other definition of CP and EP ENSO (NCP and NEP index^38^), or we use the subset of CMIP6 models that can simulate ENSO nonlinearity, both for year-round ACC and SPB (Supplementary Fig. 9, 10). The timing of the SPB (see Methods) also occurs about one month earlier as in the persistence barrier (Supplementary Fig. 7a, b), and is therefore likely contributed by the earlier persistence barrier. Increased SPB strength and earlier SPB timing of CP ENSO suggest that the future CP ENSO predictability will decline to a certain level in an earlier timing. Therefore, CP ENSO will be more unpredictable in the future.

### Understanding the enhanced CP ENSO SPB using ROM

**Fig. 3:**
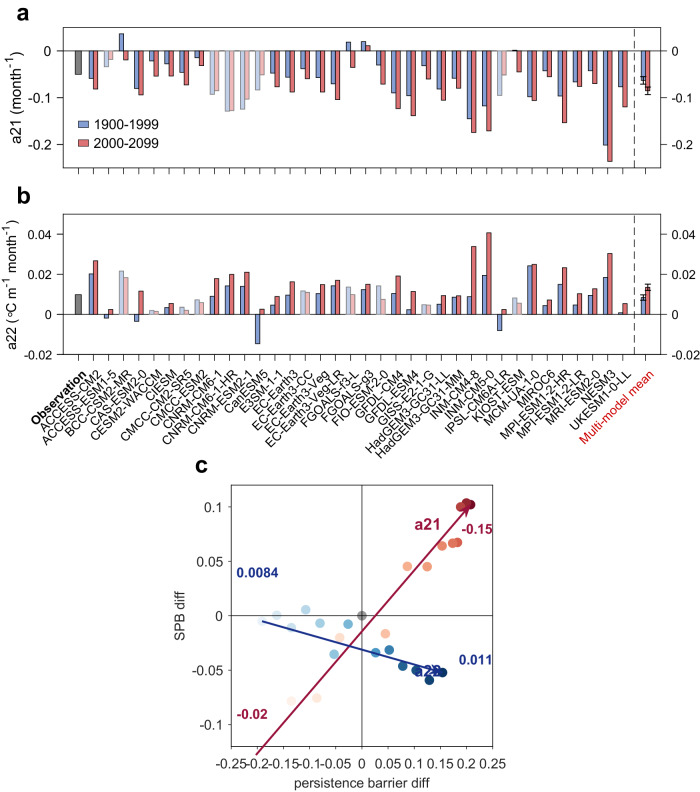
Projected changes in parameters of two-box recharge oscillation model and theirs effect of spring predictability barrier strength. **a**, **b** The mean values of (**a**) ${a}_{21}$ (unit: month^−1^) and **b** ${a}_{22}$ (unit:  °Cm^−1^) month^−1^) in different Coupled Model Intercomparison Project Phase 6 (CMIP6) models over present-day (blue bars) and future (red bars) climate. Error bars are calculated as 1.0 standard derivation of 10,000 inter-realizations of a bootstrap method (see bootstrap test in Methods). Models that are opposite to the multi-model mean result are marked in white shading. **c** The central Pacific (CP) El Niño-Southern Oscillation (ENSO) persistence barrier (x-axis) and spring predictability barrier (SPB; y-axis) difference against different damping rates (${a}_{21}$) and the coupling coefficients between T and h (${a}_{22}$). The difference is calculated relative to the case when ${a}_{21}$ is −0.05 month^−1^ and ${a}_{22}$ is 0.0098  °Cm^−1^ month^−1^. The red dots (light to dark) and arrow indicate ${a}_{21}$ ranging from −0.02 to −0.15 month^−1^. The blue dots (light to dark) and arrow indicate ${a}_{22}$ ranging from 0.0084 to 0.011  °Cm^−1^ month^−1^.

We now identify the key factors affecting the change of SPB strength and, in turn, predictability, of CP ENSO using a two-box recharge oscillation model (ROM) that is fitted to each model ENSO^40^ (see Methods). For the six parameters of the two-box ROM, only three parameters (${a}_{21}$, ${a}_{22}$ and ${a}_{31}$) show significant change under future warming climate (Supplementary Fig. 11). Physically, ${a}_{21}$ and ${a}_{31}$ are related to the growth (damping) rate of CP ENSO and thermocline depth, respectively, while ${a}_{22}$ represents the coupling effect of thermocline depth on SSTA. In total, 30 of the 36 models (83%) show decreased ${a}_{21}$ in the future period (red bars in Fig. 3a), with the multi-model mean decreased significant at the 95% confidence level; 27 of the 36 models (75%) simulate an increased ${a}_{22}$ in the future period (red bars in Fig. 3b), with the multi-model mean increased significant at the 95% confidence level. We do not discuss ${a}_{31}$ here because it does not change persistence barrier strength (Supplementary Fig. 12).

To investigate the impact of ${a}_{21}$ and ${a}_{22}$ changes on ENSO predictability in ROM, we use the prediction in the two-box ROM model, with the two parameters ${a}_{21}$ and ${a}_{22}$ changing from the present-day to future climate, and other parameters fixed (see Methods). In the perfect model framework, we use the “truth” produced by the model itself and perform ensemble forecast starting every calendar month. For the future climate, the ACC of CP ENSO is significantly reduced compared to the present-day climate when the forecast is made through the boreal spring (e.g., Supplementary Fig. 15c), leading to a stronger SPB and reduced predictability. We further compare the SPB strength of CP ENSO in the present-day and future climate. Within the 36 models, 28 models (78%) simulate an increased SPB strength of CP ENSO in the future period (Supplementary Fig. 13a), with the multi-model mean increased statistically significant at the 95% confidence level. In contrast, the change of EP ENSO predictability is insignificant (Supplementary Fig. 13b). Note here we also evaluate ENSO SPB in the CMIP6 by using this ROM (Supplementary Fig. 14; see Methods) and find that the conclusion remains the same as that in the perfect model framework. The consistent predictability change between ROM and LIM suggests the utility of ROM for understanding the decreased predictability for ENSO.

The persistence barrier and SPB strength in ROM vary with ${a}_{21}$ and ${a}_{22}$ (Fig. 3c). When ${a}_{21}$ decreases (stronger damping of the system), the strengths of the persistence barrier and SPB both strengthen (red arrow in Fig. 3c). However, when ${a}_{22}$ increases (stronger coupling effect of thermocline depth on SSTA), the strength increases for the persistence barrier, but decreases for the SPB (blue arrow in Fig. 3c). For the relationship between the persistence barrier and SPB strength in the ROM, the role of decreasing ${a}_{21}$ and increasing ${a}_{22}$ is opposite, consistent with previous findings (ref. ^25^.). This implies that the enhanced CP ENSO SPB strength under global warming is caused by the decreasing ${a}_{21}$ via increasing the CP ENSO damping rate. Note here the modulation of ${a}_{21}$ under global warming can also explain why the ACC differences peak in July instead of persisting into the subsequent wintertime (i.e., Fig. 2a). Compared to 1900–1999, ${a}_{21}$ decreases (more negative) in 2000–2099. By taking ${a}_{21}=-0.02{{{{{{\rm{month}}}}}}}^{-1}$ (Supplementary Fig. 15a) and ${a}_{21}=-0.15{{{{{{\rm{month}}}}}}}^{-1}$ (Supplementary Fig. 15b) in the ROM (other parameters unchanged), we find the maximum decrease of predictability occurs in July (Supplementary Fig. 15c). Although the annual mean damping rate, a decrease in ${a}_{21}$ leads to the lowest growth rate in the boreal spring/early summer. The system damps more quickly, and the predictability decreases most rapidly in July (Supplementary Fig. 15c).

### A possible mechanism of enhanced SPB of CP ENSO

**Fig. 4:**
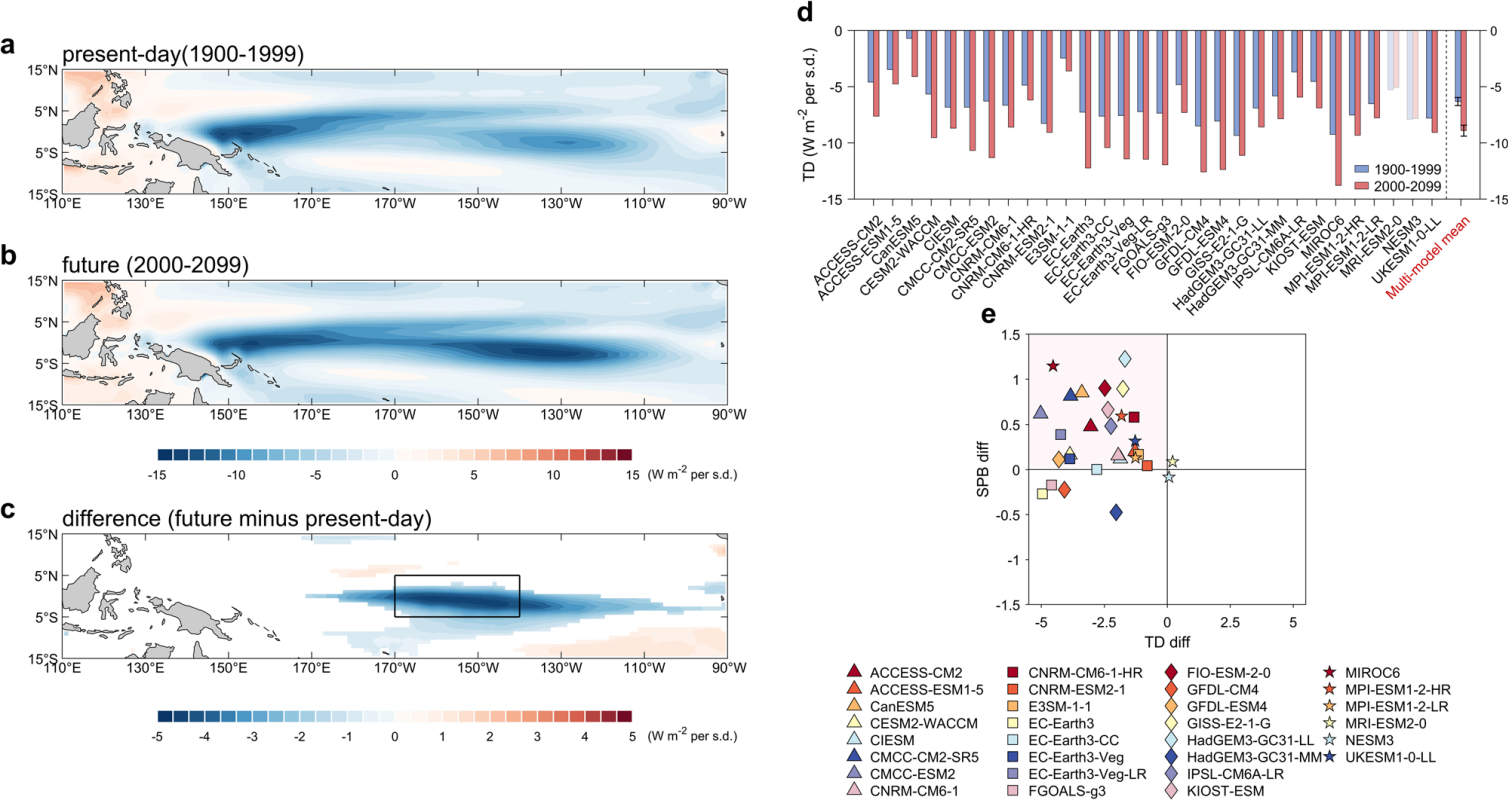
Mechanism for the projected decrease in central Pacific El Niño-Southern Oscillation predictability. **a**–**c** Multi-model mean pattern of thermodynamical damping (unit: ${{{{{\rm{W}}}}}}{{{{{{\rm{m}}}}}}}^{-2}$ ${{{{{\rm{per\; s}}}}}}.{{{{{\rm{d}}}}}}.$) in tropical Pacific under (**a**) present-day (1900–1999), **b** future climate (2000–2099), and **c** the difference between present-day and future climate. Only the difference exceeding 95% confidence level is shown. The thermodynamical damping is calculated by regressing the net surface heat flux anomalies onto the C index. **d** Comparison of central Pacific (CP) El Niño-Southern Oscillation (ENSO) thermodynamic damping over present-day (blue bars) and future (red bars). Error bars are calculated as 1.0 standard derivation of 10,000 inter-realizations of a bootstrap method (see bootstrap test in Methods). Models that are opposite to the multi-model mean result are marked in white shading. **e** Scatter plots between thermodynamic damping (*x* axis) and spring predictability barrier (SPB) difference (*y* axis; future minus present-day) for models of strengthening thermodynamical damping. The models name are listed below.

The reduced predictability of CP ENSO under greenhouse warming appears to be caused by a stronger ENSO damping rate, as encapsulated by ${a}_{21}$ in the ROM. ENSO damping rates are affected by multiple air-sea feedbacks, which include three positive feedbacks of zonal advective feedback (ZA), thermocline feedback (TH), and Ekman feedback (EK)), and two negative feedbacks of thermodynamical damping (TD) and dynamical damping (DD)^41^, as well as extratropical impact^12^. Our calculation of each feedback shows that global warming tends to enhance the three positive feedbacks^42–45^ (see also Supplementary Fig. 16 and Supplementary Table 1) as well as the North Pacific impact (Supplementary Note 2, Supplementary Fig. 17) for CP ENSO. However, these effects are overwhelmed by the even stronger increase of negative feedbacks (DD and TD terms in Supplementary Fig. 16) for CP ENSO, which leads to a net increase of the CP damping rate (${a}_{21}$ in Fig. 3a; Supplementary Fig. 16; note here the choice of the region for CP ENSO index does not change the calculated CP ENSO growth rate substantially). We further analyze the spatial pattern of TD and DD terms. The TD can be further derived by regressing the net surface heat flux anomalies onto the C index. The increased negative feedback is dominated by the increase of thermodynamic damping (TD) (Fig. 4), while the dynamic damping is not changed significantly (Supplementary Fig. 18). Therefore, TD term plays an important role in reducing CP ENSO growth rate and predictability. Compared to the present-day (Fig. 4a), the net heat flux response to C index significantly increases under greenhouse warming (Fig. 4b), especially in the equatorial central Pacific (170°W–140°W, 5°S-5°N; black box in Fig. 4c). As a quantitative measure, in the maximum TD region (black box in Fig. 4c), 93% (28 out of 30) of models show a strengthening TD, significant at the 95% confidence level (Fig. 4d). In the 28 models of increasing TD, 86% (24) models show increased SPB strength (pink area in Fig. 4e). Under greenhouse warming, the warming in SSTA^11,45^ enhances the TD via increased latent heat loss and deep clouds, acting to reduce the persistence and predictability, and in turn, increasing persistence barrier and SPB strength, of CP ENSO. The thermodynamic damping is strengthened so much that it overwhelms the enhanced predictability from the North Pacific (Supplementary Note 2).

## Discussion

In response to future greenhouse warming, we find that, across the latest CMIP6 models, predictability will be reduced for CP ENSO, but remain little changed for EP ENSO. The strength of the spring persistence barrier and SPB will be increased by 21% and 25% in the future, respectively. The predictability of CP ENSO is suppressed by the enhanced thermodynamic damping and the resulting decreasing growth rate. Furthermore, since CP ENSO frequency may be increased under greenhouse warming^11,45,46^, the CP ENSO may become the dominant mode of ENSO in the future. Our study, therefore, suggests that the prediction of CP ENSO will be more challenging in the future under global warming, which may lead to the reduced predictability of climate worldwide.

## Methods

### Reanalysis and model outputs

The monthly data (SST and depth of 20 °C isotherm) from 1958 to 2022 is obtained from the European Centre for Medium‐Range Forecasts Ocean Reanalysis System 5 (ORAS5)^47^. All monthly mean data are used after the climatological seasonal cycle, and quadratic trends are removed.

We use outputs from the CMIP6 multi-model ensemble, including monthly SST, subsurface temperature, zonal current, meridional current, vertical current, and downward net heat flux data interpolated to the same spatial 1° × 1° grid (Supplementary Table 2). These models are forced under historical anthropogenic and natural forcing up to 2014 and thereafter future greenhouse-gas forcing under the SSP585 emission scenario till 2100^48^. We compare changes in ENSO predictability between the present-day (1900–1999) and future (2000–2099) climate, covering the 200-year period. Monthly anomalies of all variables from models are obtained with reference to the monthly climatology of 1900–1999 and quadratically detrended two periods together^11,14^.

### EP and CP indices

The centers of maximum SSTA for CP and EP ENSO are different among models^11,12,37^. Here, we use the principal component (PC) time series of first two EOF modes of monthly SSTA in equatorial Pacific (5°S-5°N, 140°E-80°W) to define two indices that can represent CP and EP ENSO as in ref. ^11^. to account for model systematic errors (Supplementary Fig. 19a–d). The EOFs used to determine EP and CP ENSO were calculated using the full period. The PC time series is scaled to have a standard deviation of one. The multi-model mean patterns of the first two EOF modes reasonably resemble the observed patterns (Supplementary Fig. 19c, d vs. Supplementary Fig. 19a, b). The linear combinations of normalized PC1 and PC2 represent CP and EP ENSO, referred to as C-index ($\left({{{{{\rm{PC}}}}}}1+{{{{{\rm{PC}}}}}}2\right)/\sqrt{2}$) and E-index ($\left({{{{{\rm{PC}}}}}}1-{{{{{\rm{PC}}}}}}2\right)/\sqrt{2}$).

The nonlinearity is reflected by fitting PC1 and PC2 with the quadratic function ${{{{{\rm{PC}}}}}}2\left({{{{{\rm{t}}}}}}\right)={{{{{\rm{\alpha }}}}}}{\left[{{{{{\rm{PC}}}}}}1\left({{{{{\rm{t}}}}}}\right)\right]}^{2}+{{{{{\rm{\beta }}}}}}{{{{{\rm{PC}}}}}}1\left({{{{{\rm{t}}}}}}\right)+{{{{{\rm{\gamma }}}}}}$, with the parameter “α” (Alpha) signifying ENSO nonlinearity^37^ (Supplementary Fig. 19e). Models with a greater Alpha systematically produce a greater amplitude of E-index positive skewness and C-index negative skewness (Supplementary Fig. 19f). Therefore, Alpha is a simple yet physically sound metric for quantifying ENSO nonlinearity and we use Alpha as a criterion for selecting models. We use 28 models that produce Alpha value at least one third of the observed Alpha value (−0.32) to test the sensitivity to model selection.

### Definition of spring persistence barrier and SPB strength

The autocorrelation function (i.e. persistence) is a function of initial months *m* and lag months *t*, which can be written as *r(m,t)*^21^. ENSO spring persistence barrier strength can be defined from the autocorrelation as follows^49,50^. First, for a calendar month *m*, we identify ${t}_{B}(m)$ as the specific lag of maximum autocorrelation decline, which is calculated as the lag gradient in the time step of 1 month. The maximum gradient for initial month *m* can be calculated as

1 $${S}_{B1}\left(m\right)=\left\{\frac{r\left[m,\,{t}_{B}\left(m\right)-1\right]-r\left[m,\,{t}_{B}\left(m\right)+1\right]}{2}\right\}={\max }_{t}\left[\frac{r\left(m,\,t-1\right)-r(m,\,t+1)}{2}\right]$$

Second, the strength of the spring persistence barrier is estimated as

2 $${S}_{B}={\sum }_{m=1}^{12}{S}_{B1}(m)$$

The corresponding persistence month (${t}_{B0}(m)$) is estimated as:

3 $${t}_{B0}\left(m\right)=m+{t}_{B}(m)$$

The timing ($\overline{{t}_{B0}}$) of persistence barrier can be calculated by average the ${t}_{B0}(m)$ as:

4 $$\overline{{t}_{B0}}=\frac{1}{12}{\sum }_{m=1}^{12}{t}_{B0}\left(m\right)$$

The definition of SPB strength/timing is the same as spring persistence barrier strength/timing, but is calculated using the ACC instead of persistence.

### The two-box recharge oscillator model

The recharge/discharge paradigm also applies to CP ENSO^51^ and a new two-box recharge model for CP/EP ENSO with zonal wind stress and equatorial thermocline depth established^40^. Following the recharge-discharge framework, we adopt a two-box rechange model (ROM):

5 $$\frac{d{T}_{E}}{{dt}}={R}_{1}{T}_{E}+{a}_{12}h+{\sigma }_{E}w(t)$$

6 $$\frac{d{T}_{C}}{{dt}}={R}_{2}{T}_{C}+{a}_{22}h+{\sigma }_{c}w\left(t\right)$$

7 $$\frac{{dh}}{{dt}}={a}_{31}h+{a}_{32}\left({T}_{E}+{T}_{C}\right)+{\sigma }_{h}w\left(t\right)$$

8 $${R}_{1}={a}_{11}+{A}_{1}\sin \left({\omega }_{a}t-{\varphi }_{1}\right)$$

9 $${R}_{2}={a}_{21}+{A}_{2}\sin \left({\omega }_{a}t-{\varphi }_{2}\right)$$

Note the model used here is similar to previous studies^40^, but with some modifications. The zonal wind stress terms are omitted because their change has not effect on the strength of spring persistence barrier. ${R}_{1}$ and ${R}_{2}$ are seasonally varying as the seasonal cycle of ENSO growth rate is the cause of SPB^52^. The variable ${T}_{E}$ and ${T}_{C}$ represent the E index and C index, respectively. The h denotes the equatorial averaged thermocline depth anomalies (depth of 20 °C isotherm) in the Pacific (120°E-80°W, 5°S–5°N). ${a}_{11}$, ${a}_{21}$ and ${a}_{31}$ represent the growth (damping) rates of ${T}_{E}$, ${T}_{C}$ and h, respectively. ${a}_{12}$ and ${a}_{22}$ denote the coupling effect of h on ${T}_{E}$ and ${T}_{C}$, respectively. ${a}_{32}$ indicate the coupling effect of ${T}_{E}$ and ${T}_{C}$ on h.

All the numerical solutions of the ROM presented in this paper are calculated from the last 2000 years of a 2050-year run. The numerical models are integrated in the time step of 0.3 days. Model parameters can be estimated for observations and each model of the CMIP6 individually, using multilinear regression method. The resulting ROM model can successfully simulate the observed phase locking and SPB phenomenon for CP and EP ENSO (Supplementary Fig. 20).

We also perform ensemble forecasts using the ROM to compare the prediction skills of different initial months both in the perfect model and in the CMIP6. The parameters in the two-box ROM are obtained by the regression, which is consistent with previous study^40,53^. We regress all the parameters in simple recharge model of each CMIP6 models in present-day and future climate, respectively. To explore the roles of ${a}_{21}$ and ${a}_{22}$, other parameters (e.g., ${a}_{12}$) remain the same both in the historical and future scenarios parameters (the parameters are calculated by the averages between the two scenarios) while ${a}_{21}$ and ${a}_{22}$ are different in the two scenarios. In the perfect model framework, we use the control run as the “truth”. Then, we do ensemble forecasts every month for 400 years with 12 months forecast length to get sufficient forecast data by using these parameters. For each of the forecast ensemble members (20 members in total), a small random normal perturbation with an amplitude of 0.1 times the standard deviation of ${T}_{C}$ /${T}_{E}$ is added to the initial condition of variable ${T}_{C}$ /${T}_{E}$^54^. The CMIP6 framework is the same as the perfect model framework, except that we use E and C indices under present-day and future climate in each CMIP6 model as “truth”.

In this study, the forecast skill is quantified using the forecast ACC. The forecast ACC is defined as the temporal correlation coefficient between the ensemble mean forecast and the corresponding “truth”:

10 $${ACC}=\frac{ < {F}_{i},{O}_{i} > }{\sqrt{ < {F}_{i},{F}_{i} > < {O}_{i},{O}_{i} > }}$$

where ${F}_{i}$ is the ensemble mean forecast anomaly for forecast month or year *i*, and ${O}_{i}$ is the verifying observed anomaly. < > denotes the variance over all the months or years in verifying time series.

### Linear inverse model

In this study, we employ LIM to identify the leading feedbacks between state variables in both historical and future scenario CMIP6 simulations. Following the framework introduced in ref. ^55^, the system can be expressed as:

11 $$\frac{{{{{{\rm{d}}}}}}{{{{{\bf{x}}}}}}}{{{{{{\rm{dt}}}}}}}={{{{{\bf{Lx}}}}}}+\xi$$

where ${{{{{\bf{x}}}}}}$ is the state vector of the system, **L** represents the linear deterministic dynamic, and $\xi$ denotes the white noise forcing. **L** is determined by the lagged autocovariance matrix and the contemporaneous autocovariance matrix of ${{{{{\bf{x}}}}}}$:

12 $${{{{{\bf{L}}}}}}={{\tau }_{0}}^{-1}{{{{\mathrm{ln}}}}}\left\{{{{{{\bf{C}}}}}}\left({\tau }_{0}\right){{{{{\bf{C}}}}}}{\left(0\right)}^{-1}\right\}$$

where ${{{{{\bf{C}}}}}}\left({\tau }_{0}\right)=\left\langle {{{{{\bf{x}}}}}}(t+{\tau }_{0}){{{{{{\bf{x}}}}}}}^{T}(t)\right\rangle$ is the lagged covariance matrix of ${{{{{\bf{x}}}}}}$ at lag ${\tau }_{0}$, and ${{{{{\bf{C}}}}}}\left(0\right)=\left\langle {{{{{\bf{x}}}}}}(t){{{{{{\bf{x}}}}}}}^{T}(t)\right\rangle$ is the covariance matrix of ${{{{{\bf{x}}}}}}$. In this work, we use ${\tau }_{0}$ = 1 month. The forecast at a time lag $\tau$ from the initial calendar month $m$ can be obtained as:

13 $${{{{{{\bf{x}}}}}}}_{{{{{{\bf{f}}}}}}}\left(m+\tau \right)=\exp \left({{{{{\bf{L}}}}}}\tau \right){{{{{\bf{x}}}}}}\left(m\right)$$

The seasonal forecast skill can be quantified by calculating the ACC between ${{{{{{\bf{x}}}}}}}_{{{{{{\boldsymbol{f}}}}}}}\left(m+\tau \right)$ and the original dataset (“truth”), as per the approach described by ref. ^28^.

For the LIM presented in this work, we constructed the state vector ${{{{{\bf{x}}}}}}$ using the leading PCs of SSTA and sea surface height anomalies (SSHA). When exploring ENSO predictability within the tropical Pacific, we employ variables over to the tropical Pacific area to constitute the state vector:

14 $${{{{{{\bf{x}}}}}}}_{{TP}}=\left[\begin{array}{c}{{SST}}_{{TP}20}\\ {{SSH}}_{{TP}20}\end{array}\right]$$

where ${{SST}}_{{TP}20}$ (${{SSH}}_{{TP}20}$) contains the 12(4) leading PCs of SSTA (SSHA) in the tropical Pacific (20°S − 20°N, 140°E − 80°W). The inclusion of SSHA is motivated by research indicating that the information on tropical thermocline depth is important for improving the skill of ENSO forecasts^56^ and is crucial for capturing the diverse nature of ENSO events^57,58^. The results built upon ${{{{{{\bf{x}}}}}}}_{{TP}}$ are shown in Fig. 2. To assess the capability of LIM on simulating and predicting two types of ENSO, we constructed a stochastic time series of SST by integrating Eq. (11) with random noise/forcing following refs. ^59,60^. The regression map of the observed (reconstructed) SST onto the observed (reconstructed) E and C index is shown in Supplementary Fig. 21. Notably, the reconstructed SST exhibits similar EP and CP ENSO patterns to those observed in the original data. Additionally, the lag-covariance of SST in the tropical Pacific is also well captured by the reconstructed SST field (Supplementary Fig. 22). These results collectively indicate that our LIM approach reasonably captures the spatial and temporal characteristics of EP and CP ENSO. Here, we use 34 CMIP6 data to estimate ENSO predictability by LIM because the SSHA data is available in those models. We have implemented k-fold cross-validation to evaluate the forecast skill in the paper. A similar cross-validation method has been used in several previous studies, such as refs. ^33,56,61^. We evenly divided the 99 years of the data into 9 segments (1900–1999 or 2000–2099). When doing forecasts for each segment, we trained LIM with the rest of the years of data. After we got the forecast for the whole 99-year period, we evaluated the forecast skill by calculating the ACC between the forecast and the original data.

To further investigate the contribution of the North Pacific (e.g., NPMM) on ENSO prediction, we construct a coupled LIM framework using the state vector encompassing variables from both the tropical and northern Pacific regions:

15 $${{{{{{\bf{x}}}}}}}_{{cp}}=\left[\begin{array}{c}{{{{{{\bf{x}}}}}}}_{{{{{{\bf{TP}}}}}}}\\ {{{{{{\bf{x}}}}}}}_{{{{{{\bf{NP}}}}}}}\end{array}\right]=\left[\begin{array}{c}{{SST}}_{{TP}10}\\ {{SSH}}_{{TP}10}\\ {{SST}}_{{NP}12}\\ {{SSH}}_{{NP}12}\end{array}\right]$$

To separate the dynamics from tropical and northern Pacific, we narrow the tropical Pacific domain to (10°S − 10°N, 140°E − 80°W) and use the 12 (4) leading PCs of SSTA (SSHA) as ${{SST}}_{{TP}10}$ (${{SSH}}_{{TP}10}$). ${{SST}}_{{NP}12}$ (${{SSH}}_{{NP}12}$) contains the 6(4) leading PCs of SSTA (SSHA) in the northern Pacific (12°N − 60°N, 140°E − 100°W). The dynamics of this coupled system of tropical Pacific and northern Pacific can be decomposed by rewriting Eq. (12) as:

16 $$\frac{d{{{{{{\bf{x}}}}}}}_{{{{{{\boldsymbol{cp}}}}}}}}{{dt}}=\frac{d}{{dt}}\left[\begin{array}{c}{{{{{{\bf{x}}}}}}}_{{{{{{\bf{TP}}}}}}}\\ {{{{{{\bf{x}}}}}}}_{{{{{{\bf{NP}}}}}}}\end{array}\right]=\left[\begin{array}{cc}{{{{{{\bf{L}}}}}}}_{{{{{{\bf{TT}}}}}}} & {{{{{{\bf{L}}}}}}}_{{{{{{\bf{NT}}}}}}}\\ {{{{{{\bf{L}}}}}}}_{{{{{{\bf{TN}}}}}}} & {{{{{{\bf{L}}}}}}}_{{{{{{\bf{NN}}}}}}}\end{array}\right]\left[\begin{array}{c}{{{{{{\bf{x}}}}}}}_{{{{{{\bf{TP}}}}}}}\\ {{{{{{\bf{x}}}}}}}_{{{{{{\bf{NP}}}}}}}\end{array}\right]+\left[\begin{array}{c}{{{{{{\boldsymbol{\xi }}}}}}}_{{{{{{\bf{TP}}}}}}}\\ {{{{{{\boldsymbol{\xi }}}}}}}_{{{{{{\bf{NP}}}}}}}\end{array}\right]$$

In this coupled LIM, ${{{{{{\bf{L}}}}}}}_{{{{{{\bf{TT}}}}}}}$ and ${{{{{{\bf{L}}}}}}}_{{{{{{\bf{NN}}}}}}}$ represent the internal dynamics within the tropical and northern Pacific regions, respectively, whereas ${{{{{{\bf{L}}}}}}}_{{{{{{\bf{NT}}}}}}}$ and ${{{{{{\bf{L}}}}}}}_{{{{{{\bf{TN}}}}}}}$ represent the interaction between tropical and northern Pacific. The ENSO forecast skill achieved through this coupled LIM incorporates contributions from both tropical Pacific and northern Pacific. When ${{{{{{\bf{L}}}}}}}_{{{{{{\bf{NT}}}}}}}$ and ${{{{{{\bf{L}}}}}}}_{{{{{{\bf{TN}}}}}}}$ are set to be 0, we establish a decoupled LIM, which allows for the assessment of ENSO predictability solely from the tropical Pacific region. The differences in the forecast skill between the coupled and decoupled LIM can be used as an estimation of the northern Pacific contribution (Supplementary Fig. 17). The validity of this approach has been confirmed through previous studies that have employed it to diagnose interactions between variabilities in the tropical Pacific and other regions (e.g., refs. ^28,62^).

### Negative feedback: thermodynamical damping and dynamical damping

The term ${a}_{21}$ in two-box ROM is related to BJ index^41^ (Supplementary Table 1). The BJ index contains three positive feedbacks and two negative feedbacks, where the negative feedbacks are thermodynamical damping (TD) and dynamical damping (DD). The TD coefficient, denoted $\alpha$, is calculated via linear regression of the net downward surface heat flux anomalies onto the C index, namely

17 $${Q}_{{net}}=-\alpha \cdot C\, {index}$$

The dynamical damping represents advection due to mean zonal, meridional, and vertical currents, namely

18 $${DD}=-\left(\frac{\left\langle \bar{u}\right\rangle }{{L}_{x}}+\frac{\left\langle -2y\bar{v}\right\rangle }{{L}_{y}^{2}}+\frac{\left\langle \bar{w}\right\rangle }{{H}_{m}}\right)$$

$\bar{u}$, $\bar{v}$ and $\bar{w}$ represent climatological means of zonal, meridional, and vertical currents. ${L}_{x}$ and ${L}_{y}$ are the longitudinal and latitudinal extents of the equatorial Pacific (5°S-5°N, 120°E-90°W), respectively, and the factor −2y/${L}_{y}$ assumes that the tropical SST anomalies are Gaussian with an e-folding decay scale of ${L}_{y}$. ${H}_{m}$ is the effective depth for the vertical current that sets to 50 m.

### Statistical significance test

We use a bootstrap method to examine whether the difference in mean between sample A and sample B is statistically significant^63^. Sample A is re-sampled randomly to construct 10,000 realizations of mean standard deviation. In this random re-sampling process, any sample in sample A is allowed to be selected multiple times. The same is carried out for the sample B. The 10,000 realizations of the sum of the two standard deviations can determine 95% confidence intervals for the difference of mean between sample A and sample B. If the difference in the multi-model mean value between the two samples is greater than the sum of the two separate 10,000-realization standard deviation values, the difference is considered statistically significant above the 95% confidence level.

### Supplementary information

Supplementary Information

Peer Review File

## Data Availability

All data related to this paper can be downloaded as follows: The ORAS5 data are available at https://cds.climate.copernicus.eu/cdsapp#!/dataset/reanalysis-oras5?tab=form. The CMIP6 data can be downloaded online https://esgf-node.llnl.gov/projects/cmip6/. Source data to reproduce the figures of this paper are available on 10.5281/zenodo.11111800.

## Code availability

Code and source data for the main results is available on 10.5281/zenodo.11111800.
