# Supplementary material for: Central-Pacific El Niño-Southern Oscillation less predictable under greenhouse warming
Source: Nat Commun. 2024 May 22;15:4370. doi: 10.1038/s41467-024-48804-1 (PMC11111461; doi:10.1038/s41467-024-48804-1)
Supplement: Supplementary file 1 — Supplementary Information [file 41467_2024_48804_MOESM1_ESM.pdf]

Supplementary Information *for*

Central-Pacific El Niño-Southern Oscillation less predictable under greenhouse warming

Hui Chen[1,2], Yishuai Jin[1,2*], Zhengyu Liu[3*], Daoxun Sun[2], Xianyao Chen[1], Michael J. McPhaden[4], Antonietta Capotondi[5], Xiaopei Lin[1]

1 Frontier Science Center for Deep Ocean Multispheres and Earth System (FDOMES) and Physical Oceanography Laboratory, Ocean University of China, Qingdao, China.

2 Laoshan Laboratory, Qingdao, China.

3 Atmospheric Science Program, Department of Geography, The Ohio State University, Columbus, Ohio

4 National Oceanic and Atmospheric Administration/Pacific Marine Environmental Laboratory, Seattle, WA, USA.

5 Physical Sciences Laboratory, NOAA, Boulder, CO, USA

*Corresponding authors: Yishuai Jin (jinyishuai@126.com); Zhengyu Liu (liu.7022@osu.edu); Daoxun Sun (sdxmonkey@gmail.com)

These authors contributed equally: Hui Chen, Yishuai Jin

This file includes:

Supplementary Notes 1 to 2

Supplementary Figures 1 to 22

Supplementary Tables 1 to 2

Supplementary Note 1: CP, EP and persistence seasonality in CMIP6

Most CMIP6 models can reproduce the observational features of the CP ENSO and EP ENSO well. This can be seen in the multi-model mean of the patterns of the CP and EP ENSO in CMIP6 in comparison with observations in Supplementary Fig. 1. Most CMIP6 models also reproduce the observational features of the seasonality of the persistence, including the persistence barrier in the boreal spring, when equatorial sea surface temperature (SST) persistence exhibits a barrier that is characterized by a band of maximum decline of monthly autocorrelation of SSTA, regardless of the different initial months, as shown in the multi-model mean of the seasonal evolution of persistence in Supplementary Fig. 2.

Supplementary Note 2: The effect of North Pacific on ENSO predictability

The results derived from the coupled LIM including both the tropical and the northern Pacific reveal an intriguing contrast (Supplementary Fig. 17a). In a future warmer climate, there is a tendency for the extratropical North Pacific to enhance the predictability of CP ENSO (Supplementary Fig. 17c), which is consistent with the increased NPMM influence on ENSO suggested by ref.1. However, this effect is overwhelmed by the significant decrease in predictability by tropical internal dynamics (Supplementary Fig. 17).

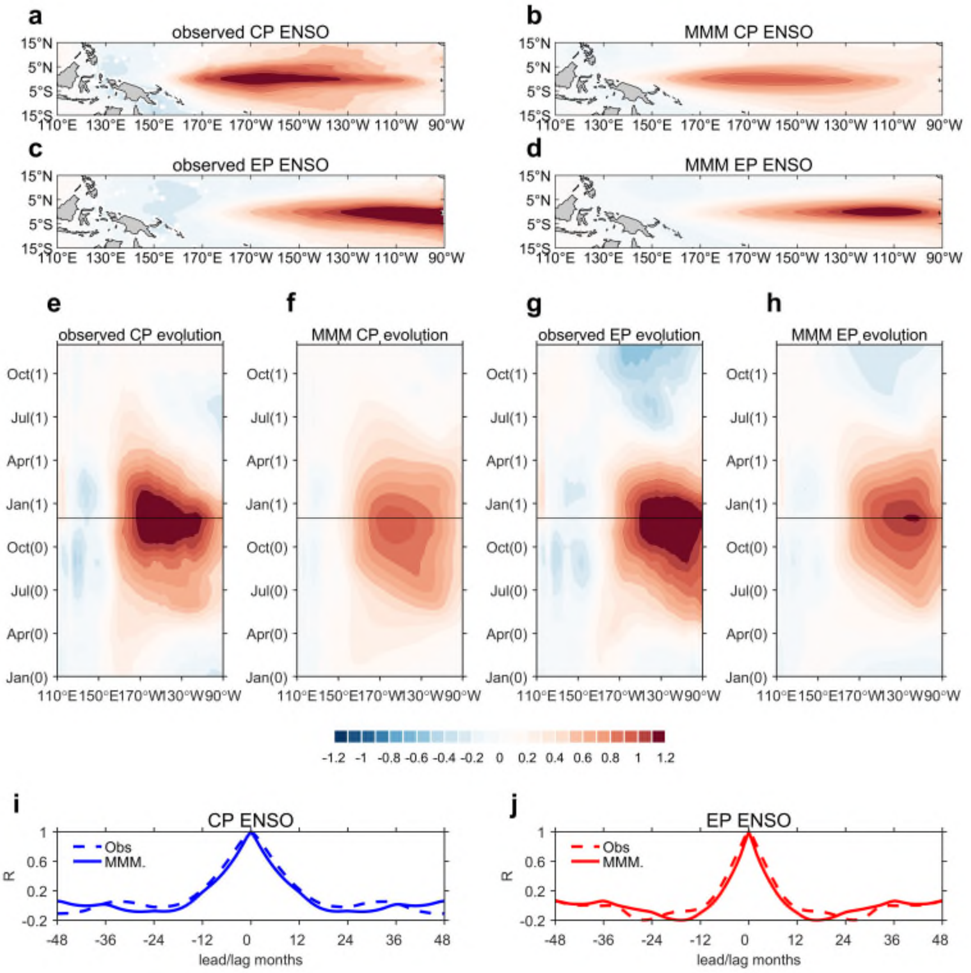

Supplementary Fig. 1 El Nino-Southern Oscillation (ENSO) sea surface temperature (SST) pattern and evolution. a, c, The observed SSTA regress onto the (**a**) C and (**c**) E index, respectively. **b**, **d**, Same as **a**, **b**, expect for the multi-model mean (MMM) results. **e**, **g**, The time evolution of observed SSTA regressed onto the (**e**) C and (**g**) E index, respectively (5°S–5°N averaged). **f**, **h**, Same as **e**, **g**, respectively, expect for the MMM results. **i**, **j**, The autocorrelation of (**i**) C and (**j**) E index for observed (dashed lines) and MMM (solid lines).

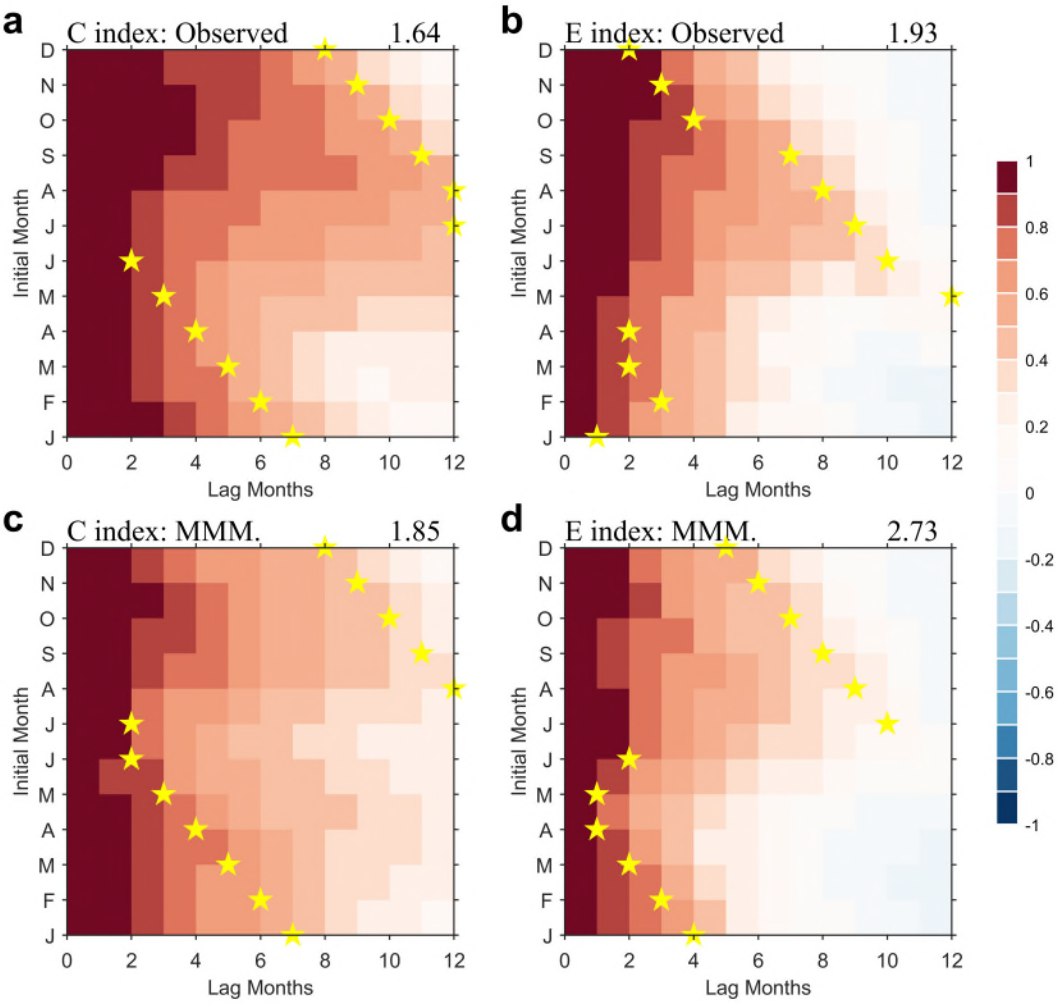

Supplementary Fig. 2 CP and EP ENSO persistence. The persistence map for (**a**) C index and (**b**) E index in observation. The yellow stars on the persistence map mark the month of maximum decline, or persistence barrier. The numbers at the top right indicate the persistence barrier strength. **c**, **d**, Same as **a**, **b**, respectively, but for multi-model mean (MMM) from 36 CMIP6 models over the whole period from 1900 to 2100.

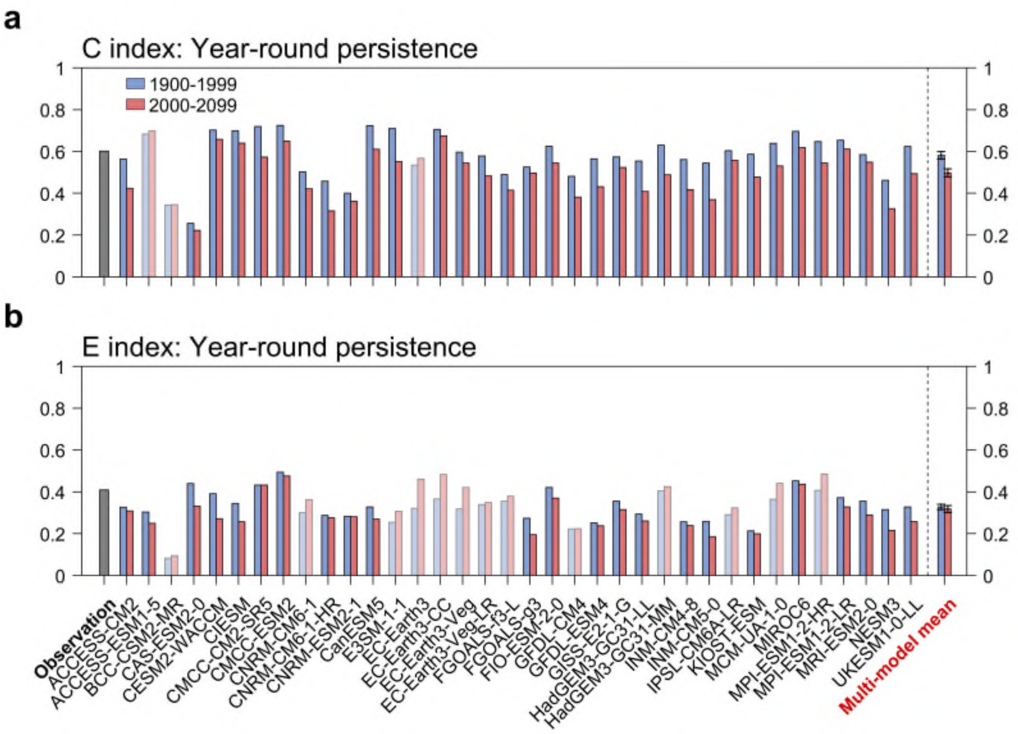

Supplementary Fig. 3 Projected changes in year-round persistence of ENSO. a, **b**, Comparison of (**a**) CP and (**b**) EP ENSO year-round SST persistence over present-day (blue bars) and future (red bars). Error bars are calculated as 1.0 standard deviation (s.d.) of 10,000 inter-realizations of a bootstrap method (see bootstrap test in Methods).

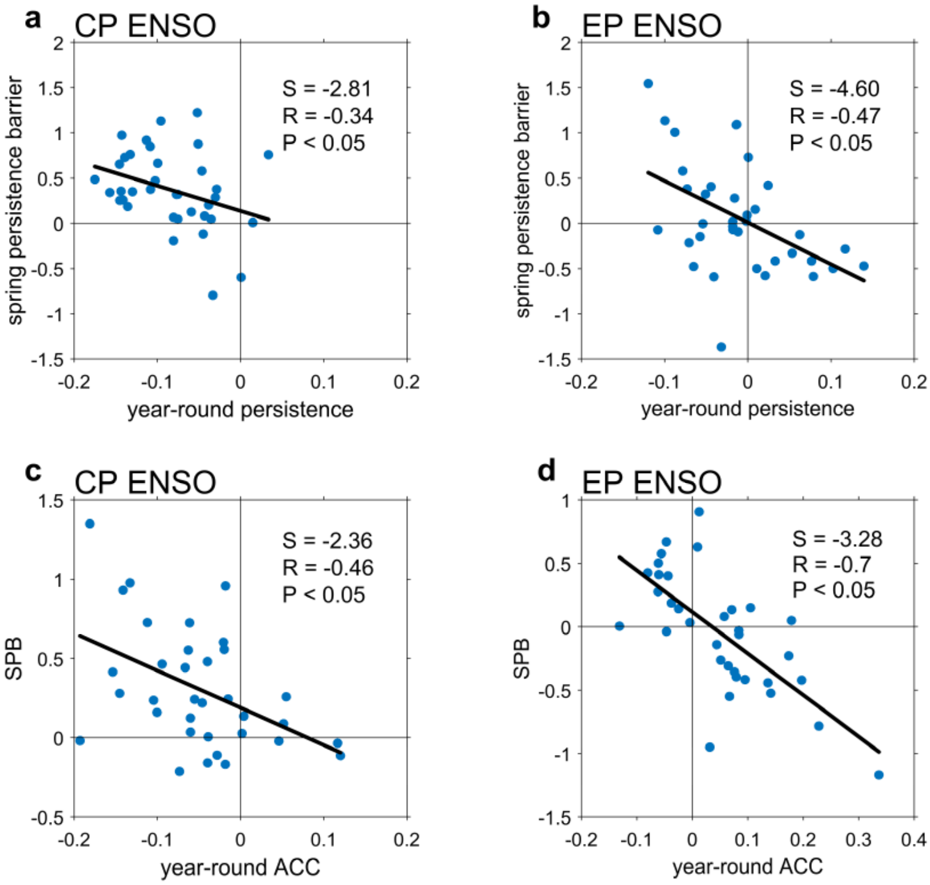

Supplementary Fig. 4 The relationship between year-round persistence/ACC and persistence barrier/SPB. **a**, **b**, The relationship of (**a**) CP ENSO and (**b**) EP ENSO year-round persistence (x-axis) and spring persistence barrier (y-axis) difference between present-day and future climate. The slope and correlation coefficient labelled on the top right. **c**, **d**, Same as **a**, **b**, but for year-round ACC and SPB.

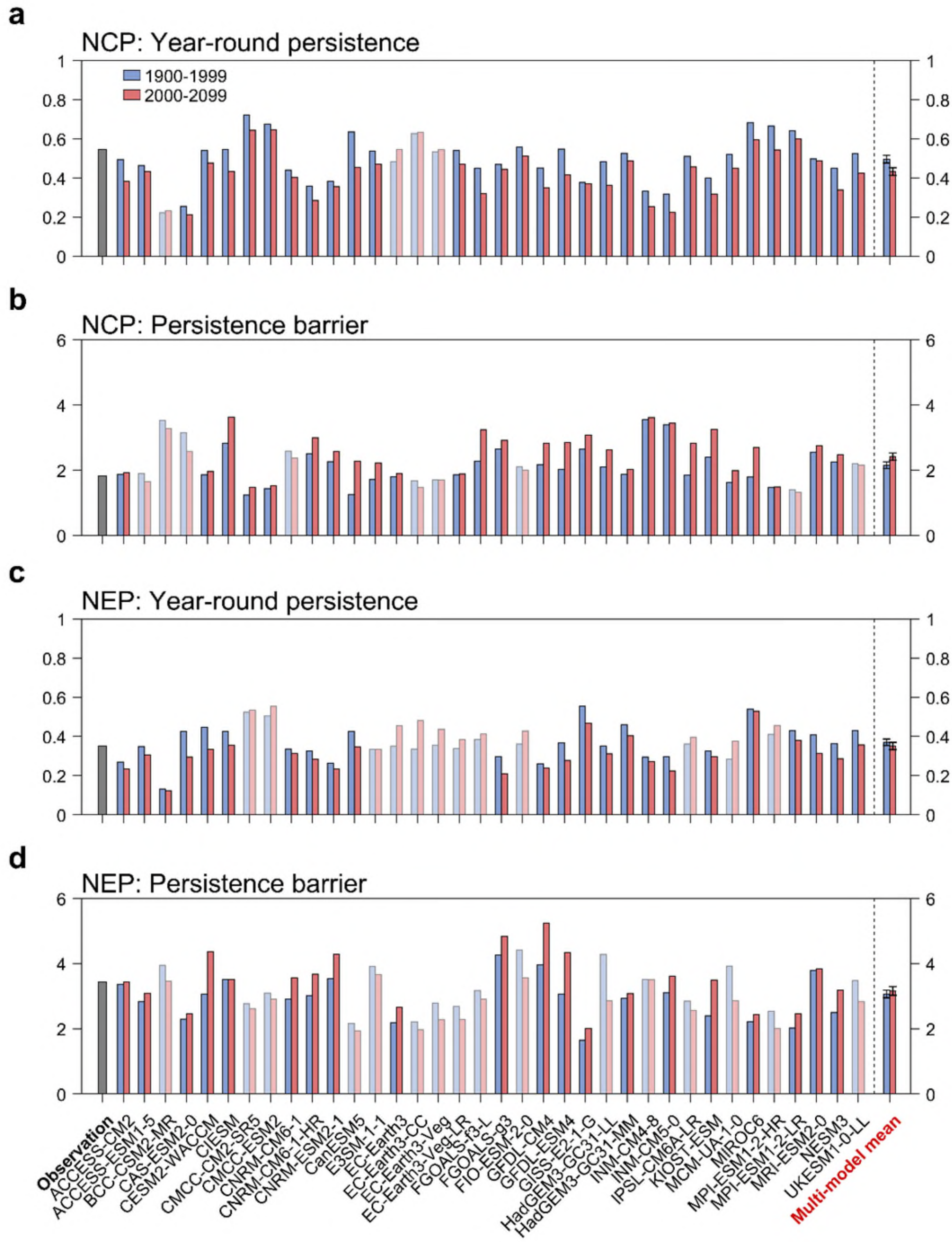

Supplementary Fig. 5 Projected change in year-round persistence and persistence barrier strength of CP and EP ENSO (by using NCP and NEP index). a, Comparison of CP ENSO year-round persistence over present-day (blue bars) and future (red bars). Error bars are calculated as 1.0 standard derivation of 10,000 inter-realizations of a bootstrap method (see bootstrap test in Methods). **b**, As in **a**, but for persistence barrier strength. **c**, **d**, Same as **a**, **b**, respectively, but for EP ENSO.

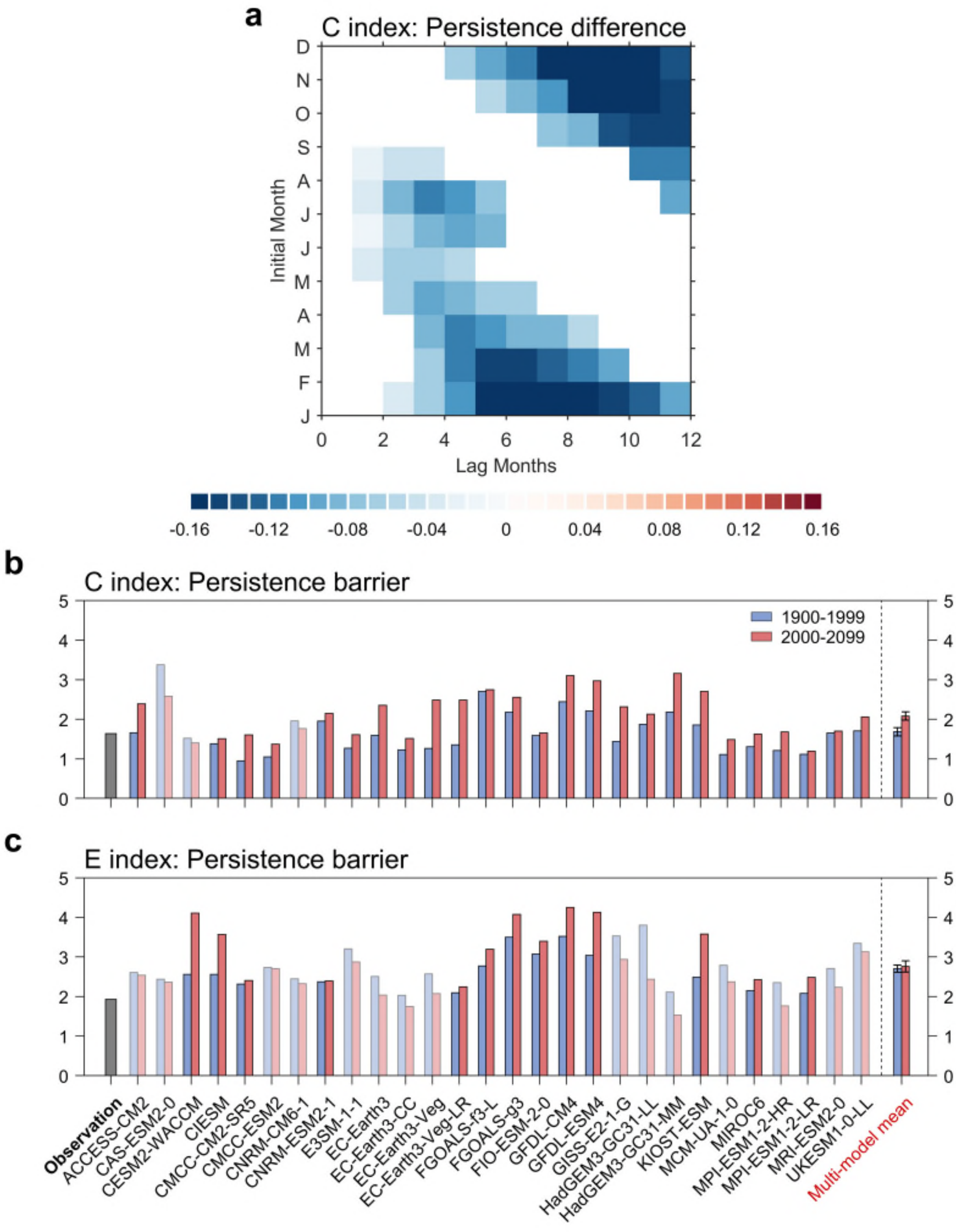

Supplementary Fig. 6 Projected changes in persistence of ENSO. Same as Fig.1, but for 28 selected model of which Alpha is at least one third of the observed value (Nonlinear relationship between PC1 and PC2 of monthly SST anomalies ($PC2(t) = \alpha[PC1(t)]^2 + \beta PC1(t) + \gamma$), with the parameter "$\alpha$" (Alpha) signifying ENSO nonlinearity; more details see "Methods").

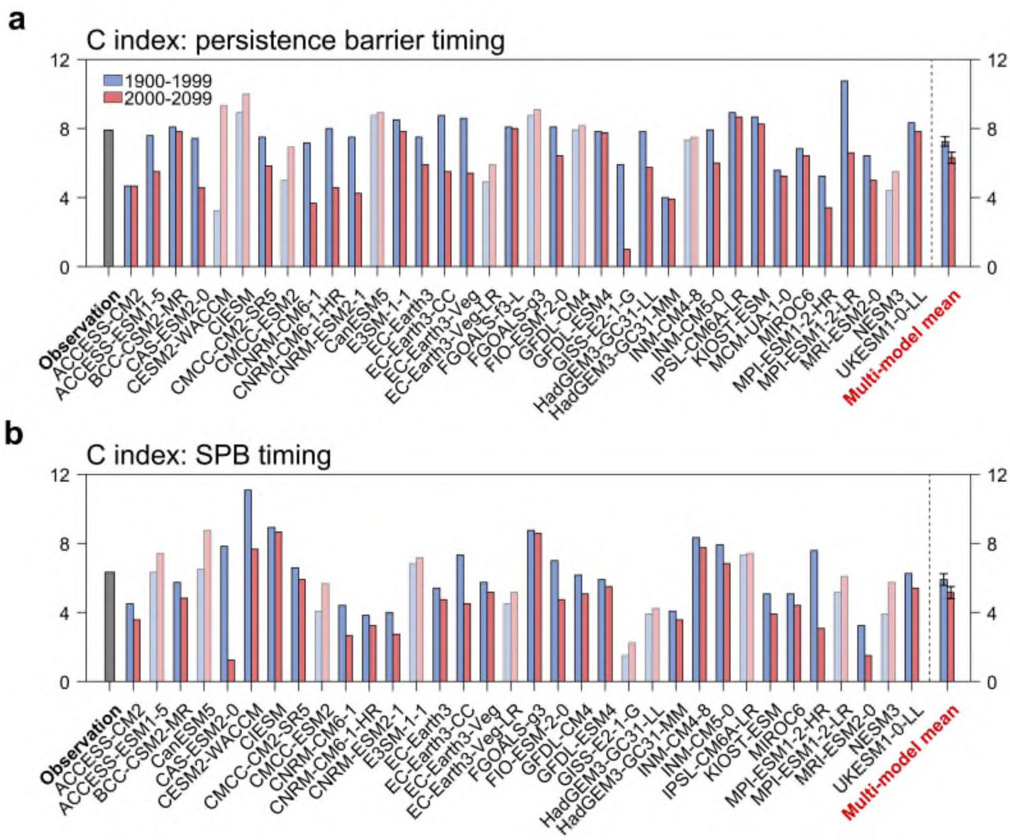

Supplementary Fig. 7 Projected change in persistence barrier timing and SPB timing of CP ENSO. a, **b**, Comparison of (**a**) persistence barrier and (**b**) SPB timing over present-day (blue bars) and future (red bars). Error bars are calculated as 1.0 standard derivation of 10,000 inter-realizations of a bootstrap method (see bootstrap test in Methods).

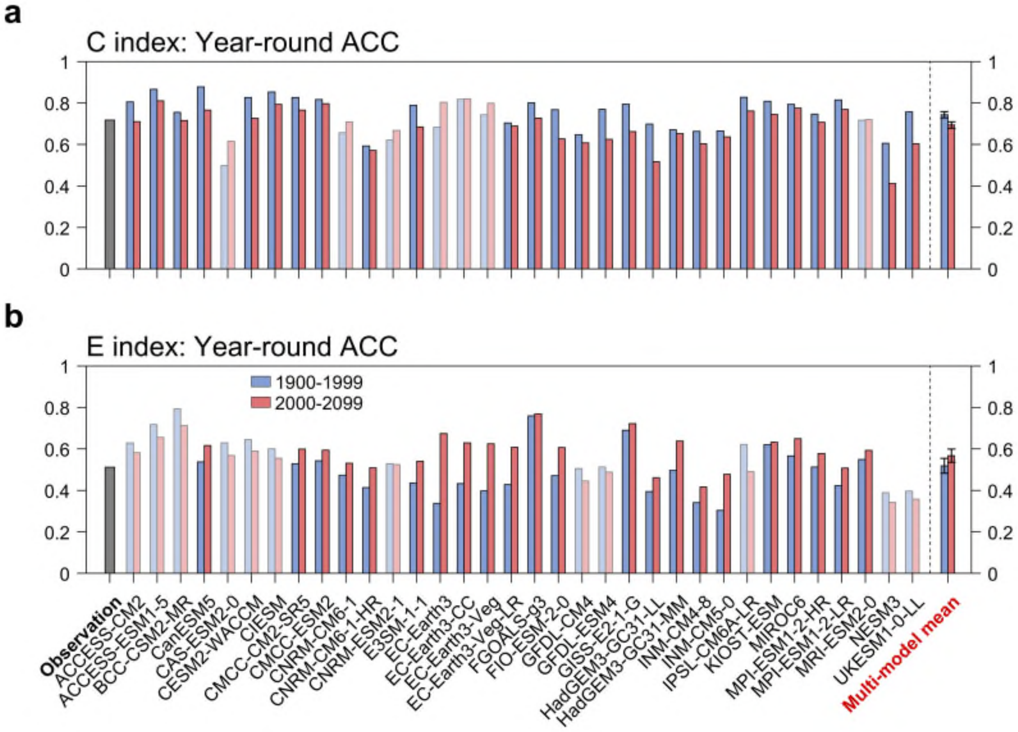

Supplementary Fig. 8 Projected change in year-round ACC of CP and EP ENSO (by using C and E index). a, Comparison of CP ENSO year-round ACC predicted by LIM over present-day (blue bars) and future (red bars). Error bars are calculated as 1.0 standard derivation of 10,000 inter-realizations of a bootstrap method (see bootstrap test in Methods). **b**, As in **a**, but for EP ENSO.

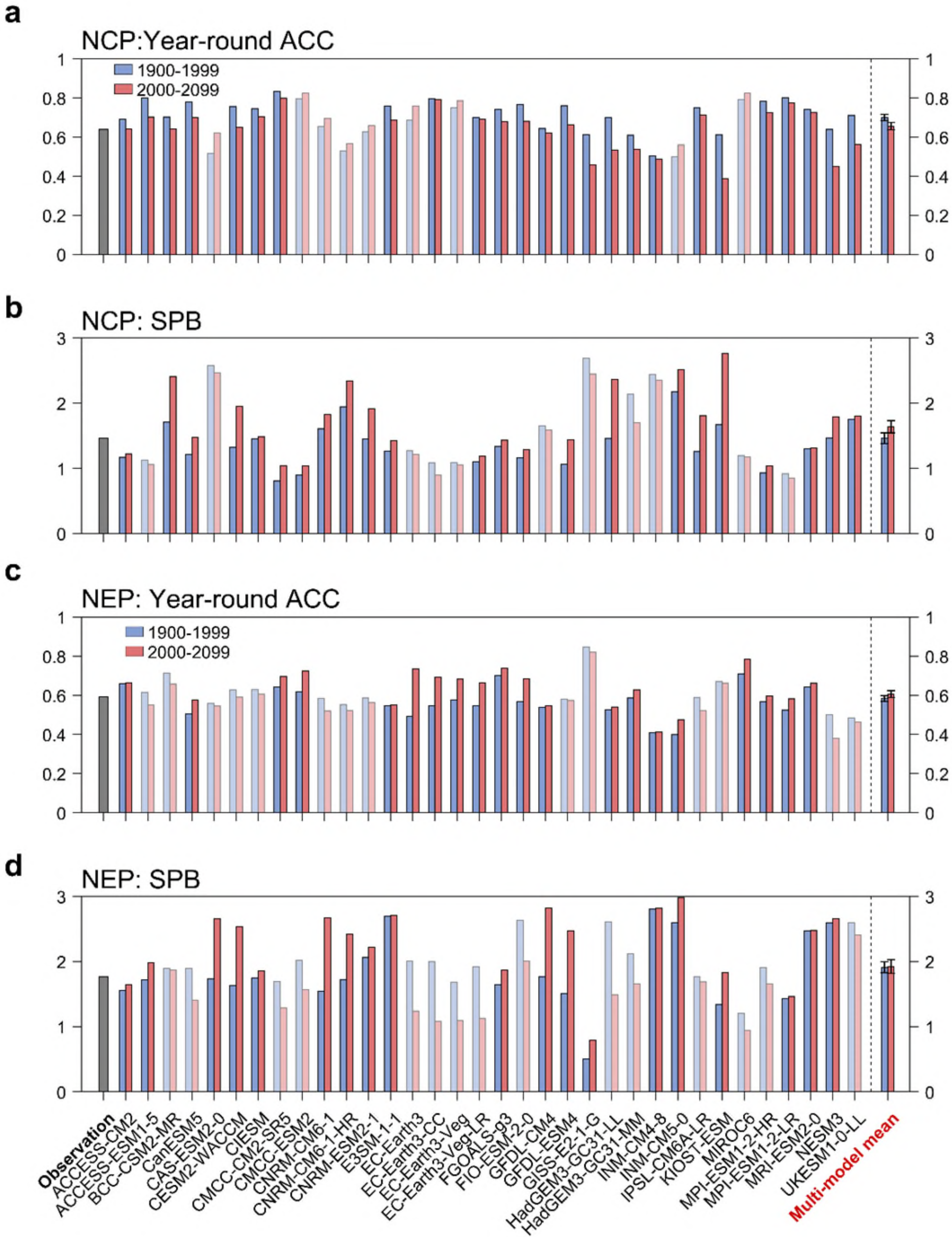

Supplementary Fig. 9 Projected change in year-round ACC and SPB strength of CP and EP ENSO (by using NCP and NEP index). a, Comparison of CP ENSO year-round ACC predicted by LIM over present-day (blue bars) and future (red bars). Error bars are calculated as 1.0 standard derivation of 10,000 inter-realizations of a bootstrap method (see bootstrap test in Methods). **b**, As in **a**, but for SPB strength. **c**, **d**, Same as **a**, **b**, respectively, but for EP ENSO.

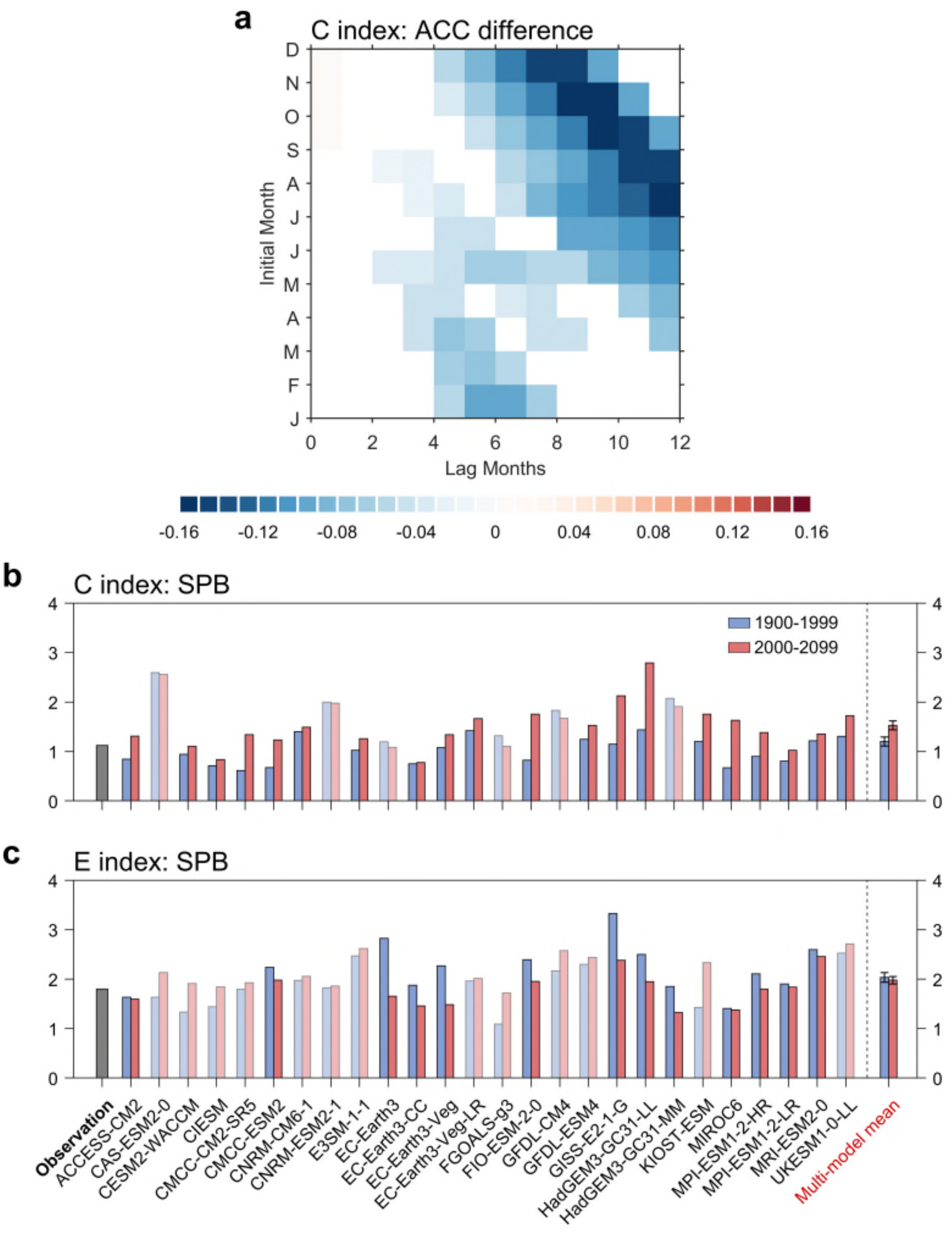

Supplementary Fig. 10 Projected change in SPB strength of ENSO. Same as Fig.2, but for 28 selected model of which Alpha is at least one third of the observed value.

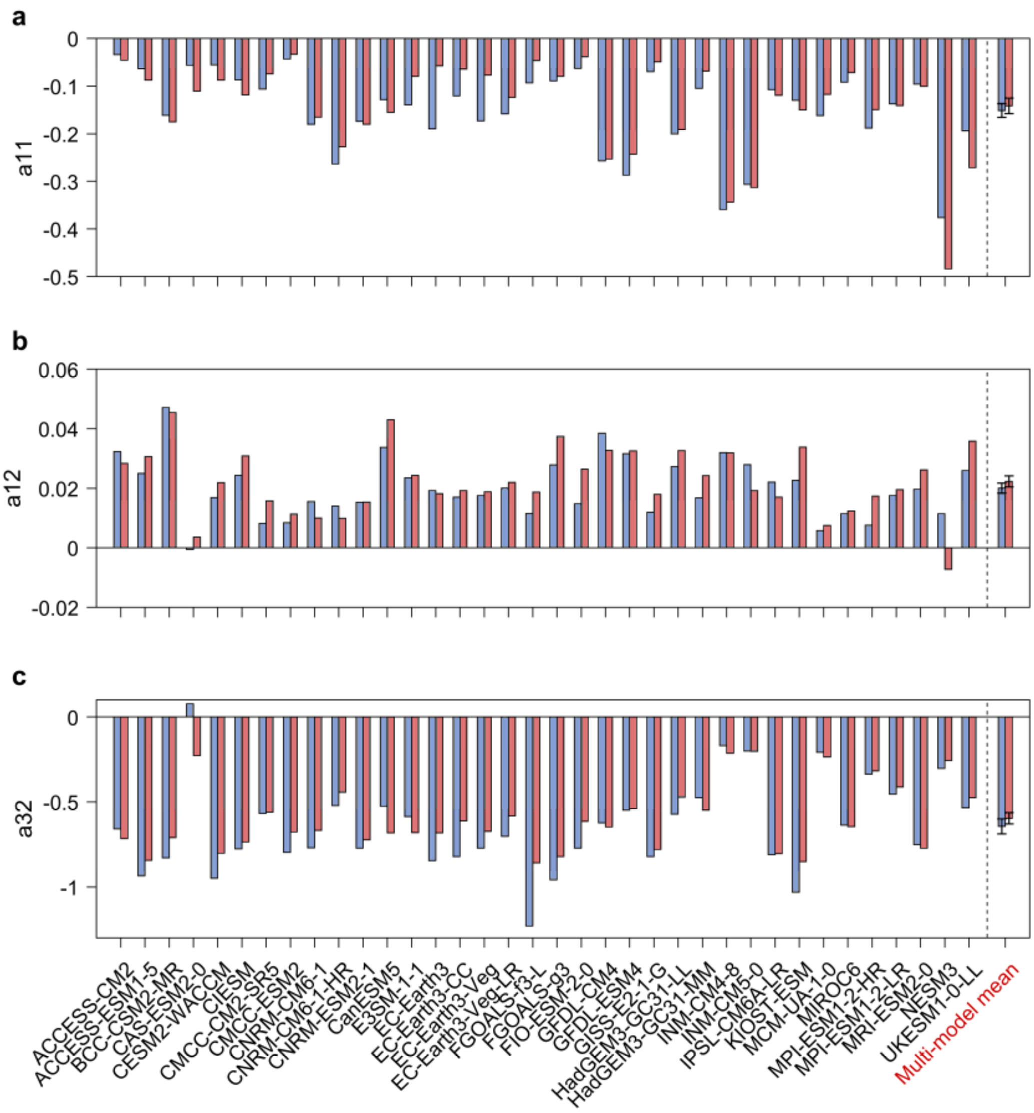

Supplementary Fig. 11 Projected changes in a11, a12, and a32 of two-box recharge model. Comparison of (**a**) a_{11} (**b**) a_{12} and (**c**) a_{32} over present-day (blue bars) and future (red bars). Error bars are calculated as 1.0 s.d. of 10,000 inter-realizations of a bootstrap method (see bootstrap test in Methods).

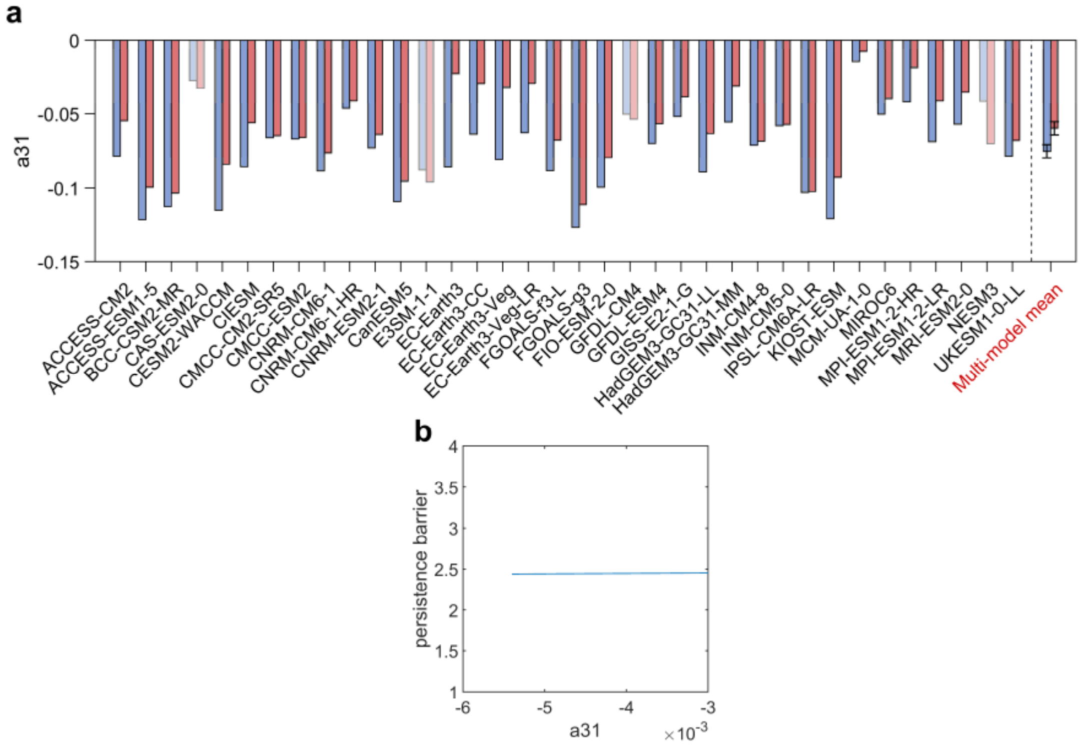

Supplementary Fig. 12 Projected changes in a31 of two-box recharge model. a, Comparison of a_{31} over present-day (blue bars) and future (red bars). Error bars are calculated as 1.0 s.d. of 10,000 inter-realizations of a bootstrap method (see bootstrap test in Methods). **b,** The spring persistence barrier strength change with a_{31}.

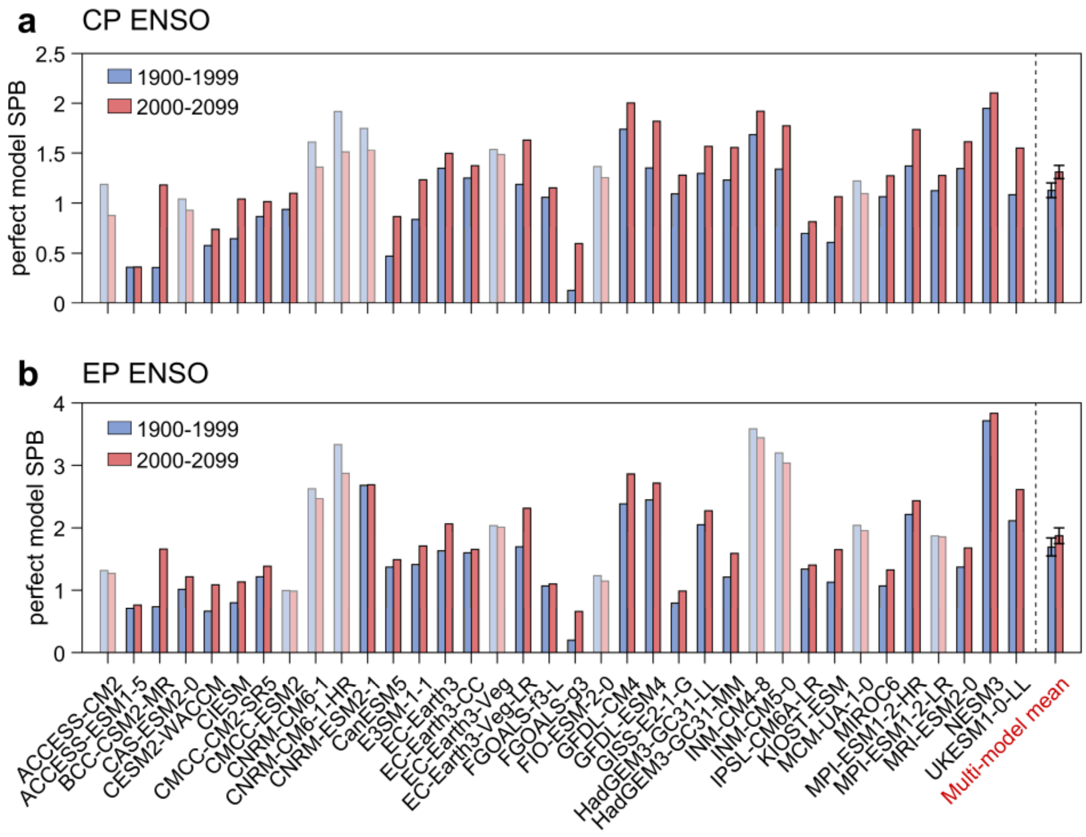

Supplementary Fig. 13 Projected changes in SPB strength in perfect model framework. Comparison of SPB strength of (**a**) CP-ENSO and (**b**) EP-ENSO over present-day (blue bars) and future (red bars). Error bars are calculated as 1.0 s.d. of 10,000 inter-realizations of a bootstrap method (see bootstrap test in Methods).

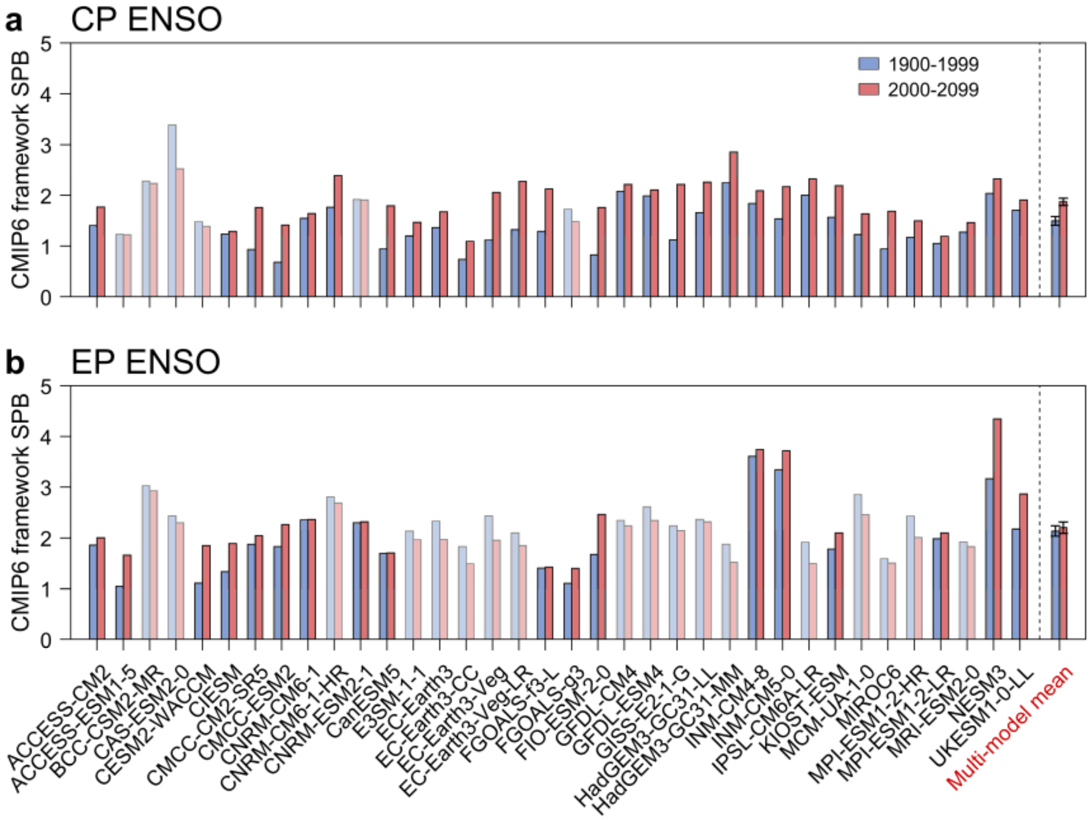

Supplementary Fig. 14 Projected changes in SPB strength in CMIP6 framework. Comparison of SPB strength of (**a**) CP-ENSO and (**b**) EP-ENSO over present-day (blue bars) and future (red bars). Error bars are calculated as 1.0 s.d. of 10,000 inter-realizations of a bootstrap method (see bootstrap test in Methods).

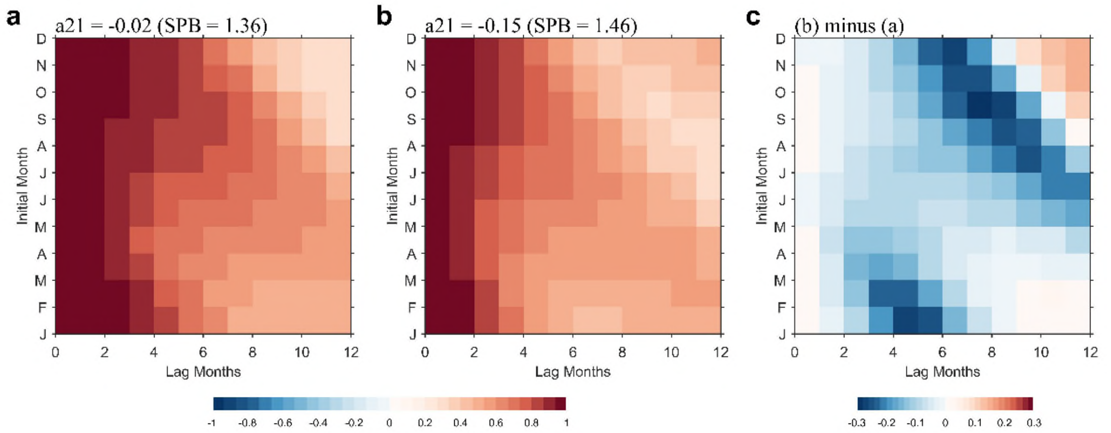

Supplementary Fig. 15 The effect of a_{21} on ENSO SPB strength. a, The ACC map obtained from ROM for CP ENSO when $a_{21} = -0.02$. **b**, As in **a**, but for $a_{21} = -0.15$. **c**, The change in ACC between **a** and **b**.

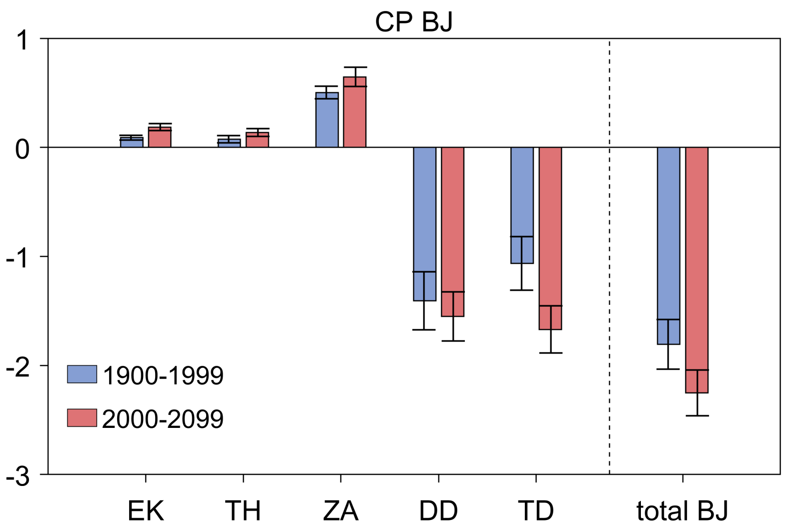

Supplementary Fig. 16 The BJ stability index. The MMM of positive and negative feedback terms from BJ stability index for present-day and future climate over CP (180°E-140°W) region. The columns from left to right indicate Ekman feedback (EK), thermocline feedback (TH), zonal advective feedback (ZA), dynamical damping (DD), thermodynamical damping (TD) and total BJ, respectively. The BJ stability indices are calculated up to 50m mixed layer. The error bars are calculated as 1.0 standard derivation of 10,000 inter-realizations of a bootstrap method (see Methods). The unit of all terms is [year^{-1}].

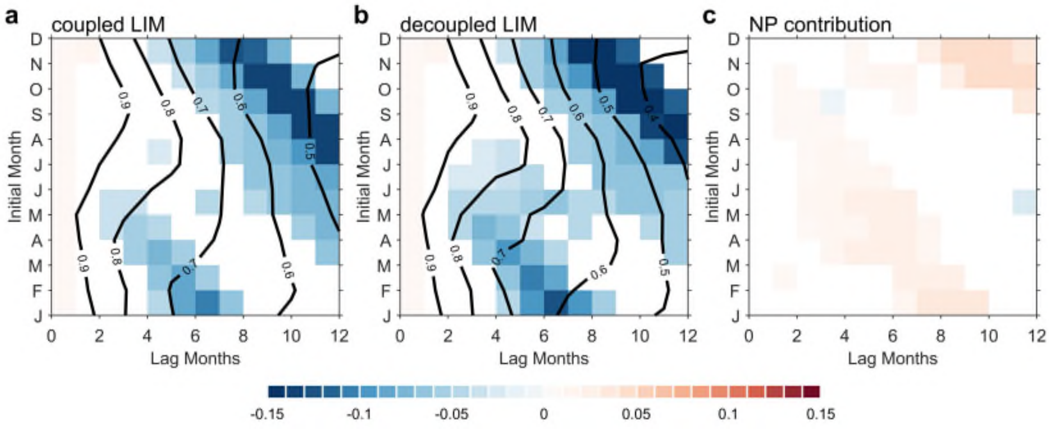

Supplementary Fig. 17 Projected change in CP-ENSO ACC predicted by LIM with and without coupling with extratropic. Same as Fig. 2a, expect for the seasonal correlation forecast skill of the C-index as a function of the initial calendar month (y-axis) and lag month (x-axis) predicted by (**a**) the tropical-extratropical coupled LIM, (**b**) the tropical-extratropical decoupled LIM and (**c**) the difference between the coupled LIM and the decoupled LIM.

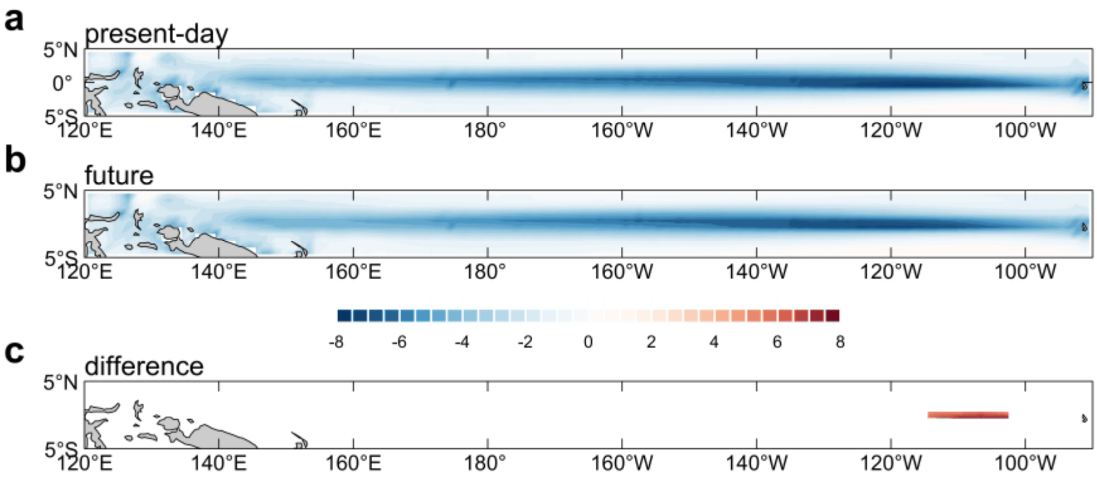

Supplementary Fig. 18 Projected changes in dynamical damping. Multi-model mean pattern of dynamical damping (unit: $year^{-1}$) in tropical Pacific under (a) present-day, (b) future climate and (c) the difference between present-day and future climate. Only the difference exceeding 95% confidence level is shown.

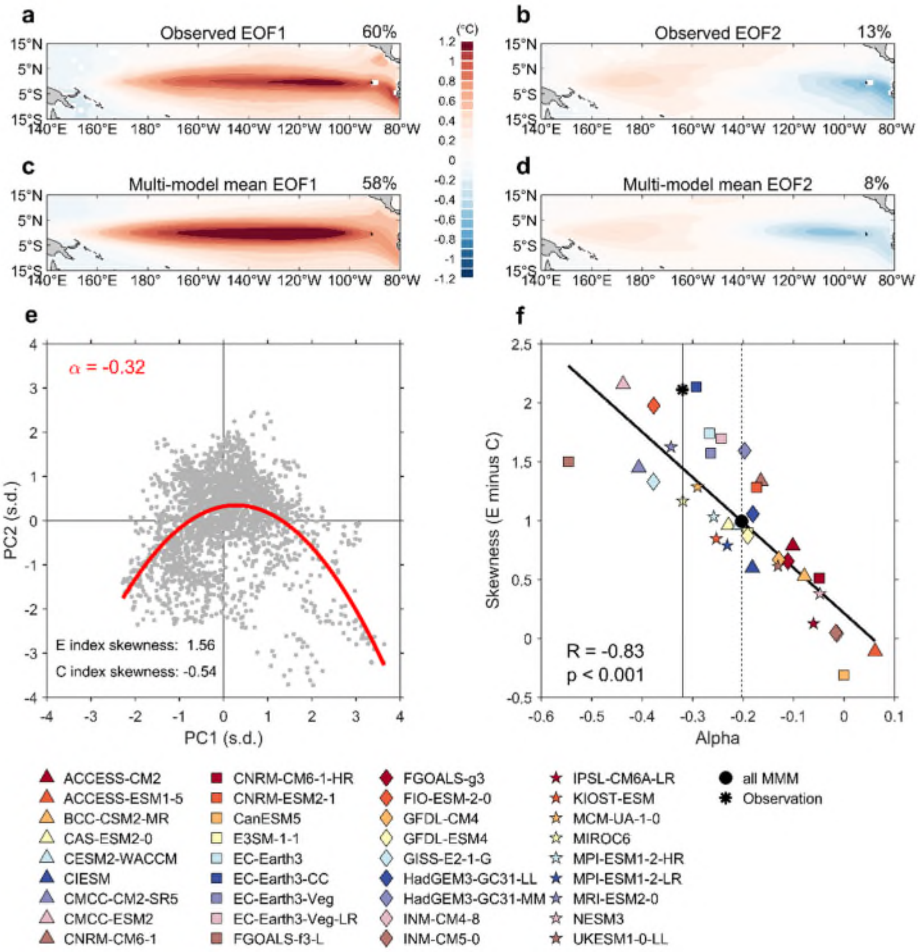

Supplementary Fig. 19 Observed and simulated ENSO nonlinearity. a, EOF1 of monthly SSTA (℃) from ORAS5 reanalysis dataset for the period of 1958-2022. **b**, As in **a**, but for EOF2. **c**, **d**, Same as **a**, **b**, respectively, but for multi-model mean (MMM) from 36 CMIP6 models over the whole period from 1900 to 2100. **e**, Nonlinear relationship between PC1 and PC2 of monthly SST anomalies (grey dots) averaged from three reanalysis products for 1958 to 2022 (ORAS5, HadISST and ERSSTv5). The red curve shows a quadratic fit $PC2(t) = \alpha[PC1(t)]^2 + \beta PC1(t) + \gamma$, with the parameter "$\alpha$" (Alpha) signifying ENSO nonlinearity. Observed values of Alpha and skewness of E index and C index are also indicated. **f**, Relationship between Alpha and magnitude of ENSO SST skewness, computed as skewness difference between E-index and C-index, for the whole period from 1900 to 2100. The vertical dashed and dash-dotted lines indicate observed and MMM values of Alpha, respectively.

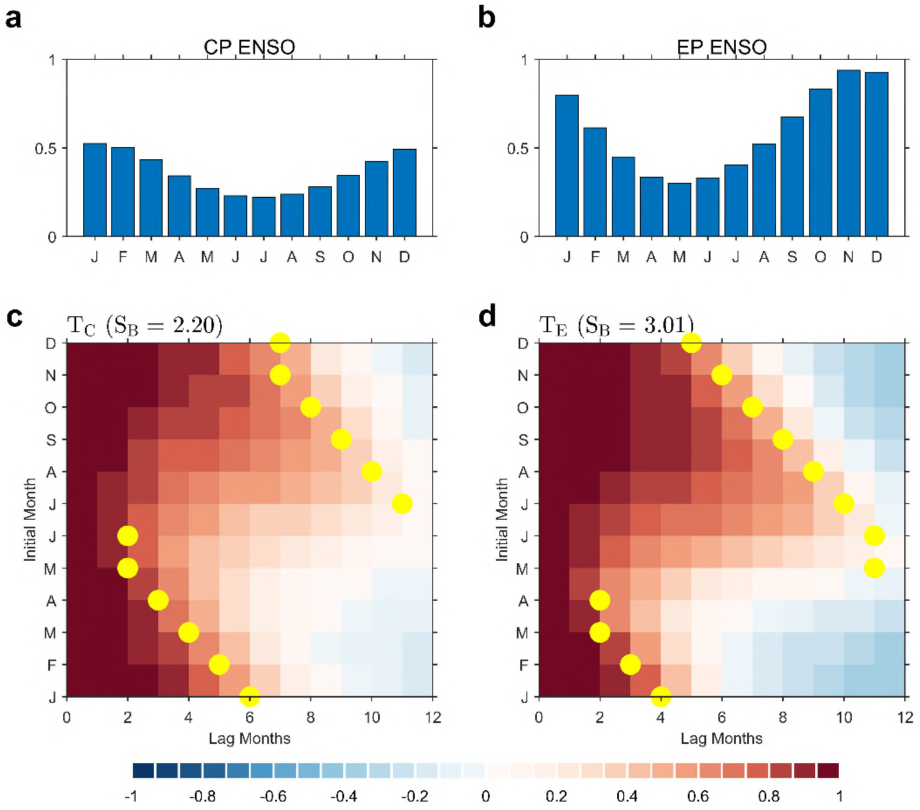

Supplementary Fig. 20 ENSO phase locking and persistence barrier in ROM. a, The CP ENSO monthly standard deviation. **c**, The persistence map for CP ENSO in control run of two-box recharge model. The yellow dots on the persistence map mark the month of maximum decline, or persistence barrier. "S_B" means the spring persistence barrier strength. **b**, **d**, Same as **a**, **c**, but for EP ENSO.

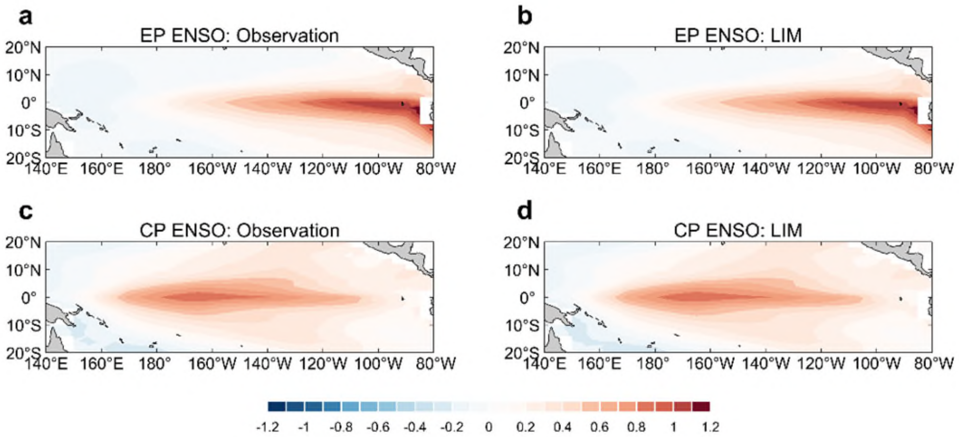

Supplementary Fig. 21 EP and CP ENSO variability captured by LIM reconstruction. Comparison of regression maps between SST and the (**a**) E and (**b**) C index in ORAS5 data. **c**, **d**, Same as **a**, **b**, but for the LIM reconstruction. The SST reconstruction is calculated by integrating equation (11) for 5700 year to get 100 stochastic samples of the ORAS5 SST.

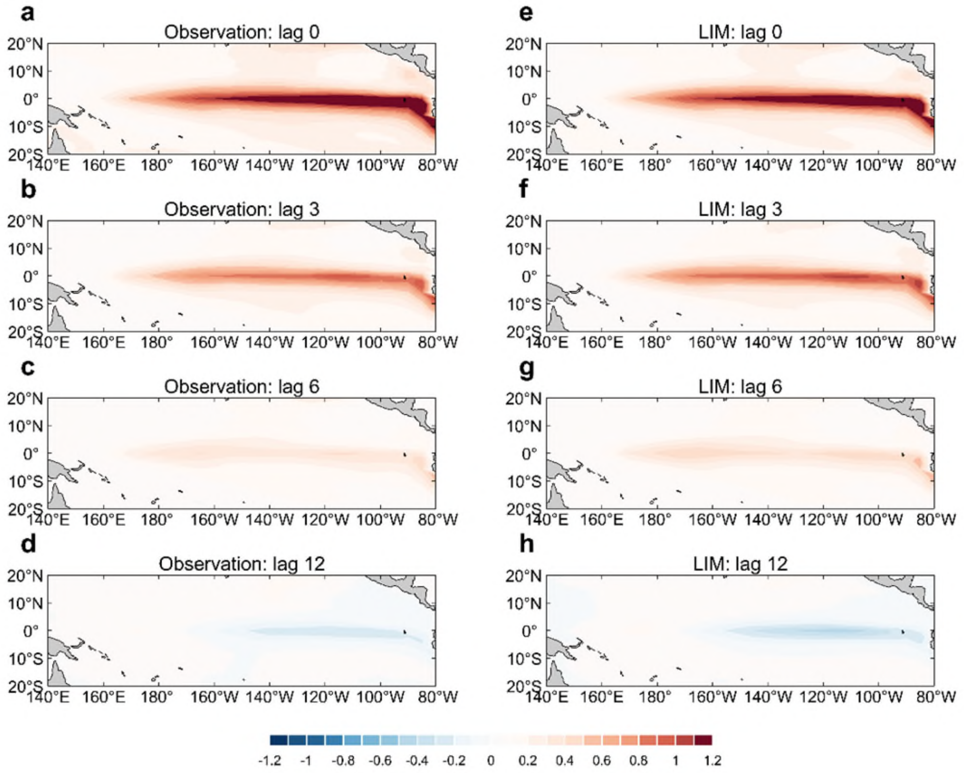

Supplementary Fig. 22 SST covariance captured by LIM reconstruction. Comparison of SST covariance with (**a**) no lag, (**b**) 3-month lag, (**c**) 6-month lag (**d**) and 12-month lag in ORAS5 data. **e-h**, Same as **a-d**, but for the LIM reconstruction.

Supplementary Table. 1 The definition of Bjerknes (BJ) stability index. BJ stability index represents the coupled stability of ocean-atmospheric interactions in the equatorial Pacific Ocean based on the recharge-oscillation theory. <A> and [A] indicate the area-averaged over local (CP) and zonal-mean (120°E-90°W), respectively.

Feedback	Equation	Components	Descriptions
Ekman feedback	$\beta_w \mu \langle -\frac{\partial \bar{T}}{\partial z} \rangle$	$[\tau_x] = \mu \langle T \rangle$<br>$\langle H(\bar{w}) w \rangle$<br>$= -\beta_w [\tau_x]$	μ: A zonal-mean wind stress response to local SST forcing<br>β_w: A local upwelling response to zonal-mean wind stress forcing
Thermocline feedback	$a_h \beta_h \mu \langle \frac{H(\bar{w})\bar{w}}{H_m} \rangle$	$[\tau_x] = \mu \langle T \rangle$<br>$\langle T_{sub} \rangle = a_h \langle h \rangle$<br>$\langle h \rangle - [h] = \beta_h [\tau_x]$	μ: A zonal-mean wind stress response to local SST forcing<br>a_h: A local subsurface ocean temperature response to local thermocline depth forcing<br>β_h: A local slope of thermocline depth response to zonal-mean wind stress forcing
Zonal advective feedback	$\beta_u \mu \langle -\frac{\partial \bar{T}}{\partial x} \rangle$	$[\tau_x] = \mu \langle T \rangle$<br>$\langle u \rangle = \beta_u [\tau_x]$	μ: A zonal-mean wind stress response to local SST forcing<br>β_u: A local zonal current response to zonal-mean wind stress forcing
Dynamical damping	$-\left(\frac{\langle \bar{u} \rangle}{L_x} + \frac{\langle -2y\bar{v} \rangle}{L_y^2} + \frac{\langle \bar{w} \rangle}{H_m} \right)$	u, v, and w represent three-dimensional (3D) ocean currents	L_x and L_y are the longitudinal and latitudinal extents, respectively, and the factor $-2y/L_y$ assumes that the tropical SST anomalies are Gaussian with an e-folding decay scale of L_y. Hm is mixed layer depth (50m)
Thermodynamical damping	$-\alpha$	$\langle Q \rangle / (\rho_0 C_p H)$<br>$= -\alpha \langle T \rangle$	α: linear regression of the net downward surface heat flux anomalies onto the SSTA<br>ρ_0: the reference density of sea water (1025 kg m^{-3})<br>C_p: the specific heat of seawater at constant pressure<br>(3994 J kg^{-1} $°C^{-1}$)

Supplementary Table 2 CMIP6 models list

Model number	Model name	Ensemble member	Data availiable
1	ACCESS-CM2	r1i1p1f1	tos, zos, thetao, tauu, hfds
2	ACCESS-ESM1-5	r1i1p1f1	tos, zos, thetao, tauu, uo, vo, wo, hfds
3	BCC-CSM2-MR	r1i1p1f1	tos, zos, thetao, tauu
4	CanESM5	r1i1p1f1	tos, zos, thetao, tauu, uo, vo, wo, hfds
5	CAS-ESM2-0	r1i1p1f1	tos, zos, thetao, tauu
6	CESM2-WACCM	r1i1p1f1	tos, zos, thetao, tauu, hfds
7	CIESM	r1i1p1f1	tos, zos, thetao, tauu, hfds
8	CMCC-CM2-SR5	r1i1p1f1	tos, zos, thetao, tauu, hfds
9	CMCC-ESM2	r1i1p1f1	tos, zos, thetao, tauu, uo, vo, wo, hfds
10	CNRM-CM6-1	r1i1p1f2	tos, zos, thetao, tauu, hfds
11	CNRM-CM6-1-HR	r1i1p1f2	tos, zos, thetao, tauu, hfds
12	CNRM-ESM2-1	r1i1p1f2	tos, zos, thetao, tauu, uo, vo, wo, hfds
13	E3SM-1-1	r1i1p1f1	tos, zos, thetao, tauu, uo, vo, wo, hfds
14	EC-Earth3	r1i1p1f1	tos, zos, thetao, tauu, hfds
15	EC-Earth3-CC	r1i1p1f1	tos, zos, thetao, tauu, hfds
16	EC-Earth3-Veg	r1i1p1f1	tos, zos, thetao, tauu, hfds
17	EC-Earth3-Veg-LR	r1i1p1f1	tos, zos, thetao, tauu, uo, vo, wo, hfds
18	FGOALS-f3-L	r1i1p1f1	tos, thetao, tauu
19	FGOALS-g3	r1i1p1f1	tos, zos, thetao, tauu, uo, vo, wo, hfds
20	FIO-ESM-2-0	r1i1p1f1	tos, zos, thetao, tauu, uo, vo, wo, hfds
21	GFDL-CM4	r1i1p1f1	tos, zos, thetao, tauu, hfds
22	GFDL-ESM4	r1i1p1f1	tos, zos, thetao, tauu, hfds
23	GISS-E2-1-G	r1i1p1f2	tos, zos, thetao, tauu, uo, vo, wo, hfds
24	HadGEM3-GC31-LL	r1i1p1f3	tos, zos, thetao, tauu, hfds
25	HadGEM3-GC31-MM	r1i1p1f3	tos, zos, thetao, tauu, hfds
26	INM-CM4-8	r1i1p1f1	tos, zos, thetao, tauu
27	INM-CM5-0	r1i1p1f1	tos, zos, thetao, tauu
28	IPSL-CM6A-LR	r1i1p1f1	tos, zos, thetao, tauu, uo, vo, wo, hfds
29	KIOST-ESM	r1i1p1f1	tos, zos, thetao, tauu, hfds
30	MCM-UA-1-0	r1i1p1f2	tos, thetao, tauu
31	MIROC6	r1i1p1f1	tos, zos, thetao, tauu, uo, vo, wo, hfds
32	MPI-ESM1-2-HR	r1i1p1f1	tos, zos, thetao, tauu, uo, vo, wo, hfds
33	MPI-ESM1-2-LR	r1i1p1f1	tos, zos, thetao, tauu, hfds
34	MRI-ESM2-0	r1i1p1f1	tos, zos, thetao, tauu, hfds
35	NESM3	r1i1p1f1	tos, zos, thetao, tauu, uo, vo, wo, hfds
36	UKESM1-0-LL	r1i1p1f2	tos, zos, thetao, tauu, hfds

Supplementary References

1. Jia, F., Cai, W., Gan, B., Wu, L. & Di Lorenzo, E. Enhanced North Pacific impact on El Niño/Southern Oscillation under greenhouse warming. *Nat. Clim. Change* **11**, 840–847 (2021).
